# Epigenomic signatures in liver and blood of Wilson disease patients include hypermethylation of liver-specific enhancers

**DOI:** 10.1186/s13072-019-0255-z

**Published:** 2019-02-01

**Authors:** Charles E. Mordaunt, Dorothy A. Kieffer, Noreene M. Shibata, Anna Członkowska, Tomasz Litwin, Karl-Heinz Weiss, Yihui Zhu, Christopher L. Bowlus, Souvik Sarkar, Stewart Cooper, Yu-Jui Yvonne Wan, Mohamed R. Ali, Janine M. LaSalle, Valentina Medici

**Affiliations:** 1 Department of Medical Microbiology and Immunology, Genome Center, and MIND Institute, University of California Davis, Davis, CA USA; 2 Division of Gastroenterology and Hepatology, Department of Internal Medicine, University of California Davis, Sacramento, CA USA; 3 2nd Department of Neurology, Institute of Psychiatry and Neurology, Warsaw, Poland; 4 Department of Internal Medicine IV, University Hospital Heidelberg, Heidelberg, Germany; 5 California Pacific Medical Center, San Francisco, CA USA; 6 Department of Pathology and Laboratory Medicine, University of California Davis, Sacramento, CA USA; 7 Department of Surgery, University of California Davis, Sacramento, CA USA

**Keywords:** Wilson disease, Epigenetics, Copper, DNA methylation, Whole-genome bisulfite sequencing, Chromatin, Liver, Blood, Biomarker, Enhancer

## Abstract

**Background:**

Wilson disease (WD) is an autosomal recessive disease caused by mutations in *ATP7B* encoding a copper transporter. Consequent copper accumulation results in a variable WD clinical phenotype involving hepatic, neurologic, and psychiatric symptoms, without clear genotype–phenotype correlations. The goal of this study was to analyze alterations in DNA methylation at the whole-genome level in liver and blood from patients with WD to investigate epigenomic alterations associated with WD diagnosis and phenotype. We used whole-genome bisulfite sequencing (WGBS) to examine distinct cohorts of WD subjects to determine whether DNA methylation could differentiate patients from healthy subjects and subjects with other liver diseases and distinguish between different WD phenotypes.

**Results:**

WGBS analyses in liver identified 969 hypermethylated and 871 hypomethylated differentially methylated regions (DMRs) specifically identifying patients with WD, including 18 regions with genome-wide significance. WD-specific liver DMRs were associated with genes enriched for functions in folate and lipid metabolism and acute inflammatory response and could differentiate early from advanced fibrosis in WD patients. Functional annotation revealed that WD-hypermethylated liver DMRs were enriched in liver-specific enhancers, flanking active liver promoters, and binding sites of liver developmental transcription factors, including Hepatocyte Nuclear Factor 4 alpha (HNF4A), Retinoid X Receptor alpha (RXRA), Forkhead Box A1 (FOXA1), and FOXA2. DMRs associated with WD progression were also identified, including 15 with genome-wide significance. However, WD DMRs in liver were not related to large-scale changes in proportions of liver cell types. DMRs detected in blood differentiated WD patients from healthy and disease control subjects, and distinguished between patients with hepatic and neurologic WD manifestations. WD phenotype DMRs corresponded to genes enriched for functions in mental deterioration, abnormal B cell physiology, and as members of the polycomb repressive complex 1 (PRC1). 44 DMRs associated with WD phenotype tested in a small validation cohort had a predictive value of 0.9.

**Conclusions:**

We identified a disease-mechanism relevant epigenomic signature of WD that reveals new insights into potential biomarkers and treatments for this complex monogenic disease.

**Electronic supplementary material:**

The online version of this article (10.1186/s13072-019-0255-z) contains supplementary material, which is available to authorized users.

## Background

Wilson disease (WD) is an autosomal recessive disease caused by copper accumulation mainly in the liver and in the brain as a result of mutations affecting the copper transporter gene, *ATPase copper transporting beta* (*ATP7B*). In healthy individuals, ATP7B contributes to copper trafficking within the hepatocyte and is required for copper excretion into the biliary tract [1, 2]. However, while the genetic basis is now better understood, WD still represents a clinically challenging and often unrecognized condition due to incomplete understanding of in-depth pathogenic mechanisms, lack of a gold standard diagnostic test, and ultimately limited treatment options [3]. The clinical presentation includes hepatic, neurologic, and psychiatric manifestations. The lack of a clear correlation between DNA sequence mutations and clinical presentation can be attributed to the presence of more than 500 mutations combined to impair ATP7B copper transporter activity, and the potential presence of concomitant modifier genes. The most striking evidence that factors aside from genetic mutations affect the phenotype is derived from multiple case reports describing monozygotic twins affected by WD but presenting different phenotypes [4, 5].

DNA methylation is a reversible epigenetic modification that is affected by both genetic and non-genetic factors such as nutrition and toxin exposure, and acts at the interface between genetics and the environment. Nutritional factors affect methionine metabolism and the global availability of methyl groups for DNA and histone methylation. Animal models of WD show changes in methionine metabolism [6, 7] and gene transcript levels in response to dietary provision of methyl donors [8]. In particular, copper is known to interfere with expression and activity of the enzyme *S*-adenosylhomocysteine hydrolase (SAHH) which in turn can affect *S*-adenosylhomocysteine (SAH) levels and DNA methyltransferase activity and expression [9].

DNA methylation can be altered at individual loci by transcription factor-mediated recruitment of DNA methyltransferases and demethylases, affecting future transcription factor binding efficiencies [10]. Methylation at the gene promoter is negatively associated with gene expression, while gene body methylation is positively associated. In patients with common liver conditions, changes in gene-specific DNA methylation patterns have been observed in both blood and liver and have been correlated with different stages of liver disease severity [11]. Although liver and brain are the most affected tissues in WD, copper also increases in blood plasma [12]. An increased humoral immune response, a decreased cell-mediated immune response, and increases in gamma delta + T cells have been found in the blood of WD patients, suggesting that both liver and blood may have characteristic methylation signatures in WD [13, 14].

There is strong evidence supporting the involvement of alterations in methionine metabolism in the pathogenesis of WD in animal models and indirect evidence supporting a role for the same mechanisms in the onset and progression of this condition in humans [15]. In the present study, we explored the hypothesis that DNA methylation patterns both in liver and in the blood can differentiate patients with WD of various phenotypic presentation from healthy subjects and from subjects affected by other liver conditions, including non-alcoholic fatty liver disease (NAFLD) and primary sclerosing cholangitis (PSC), with the ultimate objective of identifying new insights into pathogenic mechanisms, diagnostic markers, and targets for treatment.

## Results

### Clinical features of liver samples

A total of 21 liver samples were available for methylome analysis, including 6 from healthy controls (HC) undergoing bariatric surgery with normal liver histology, 5 from subjects with NAFLD (disease controls, DC), and 10 from patients with WD, including 5 percutaneous liver biopsies and 5 from explanted livers, both from cirrhosis and acute liver failure patients (Additional file 1: Table S1, Additional file 2: Table S2). The median age for all subjects was 44 years old (range 22–72, not different between the three groups). HC had higher BMIs compared to WD patients since samples were derived from bariatric surgery patients. WD patients presented significantly more advanced stage of fibrosis compared to the other subjects, as expected.

### DMRs in liver distinguish WD patients from both healthy and disease controls

**Fig. 1:**
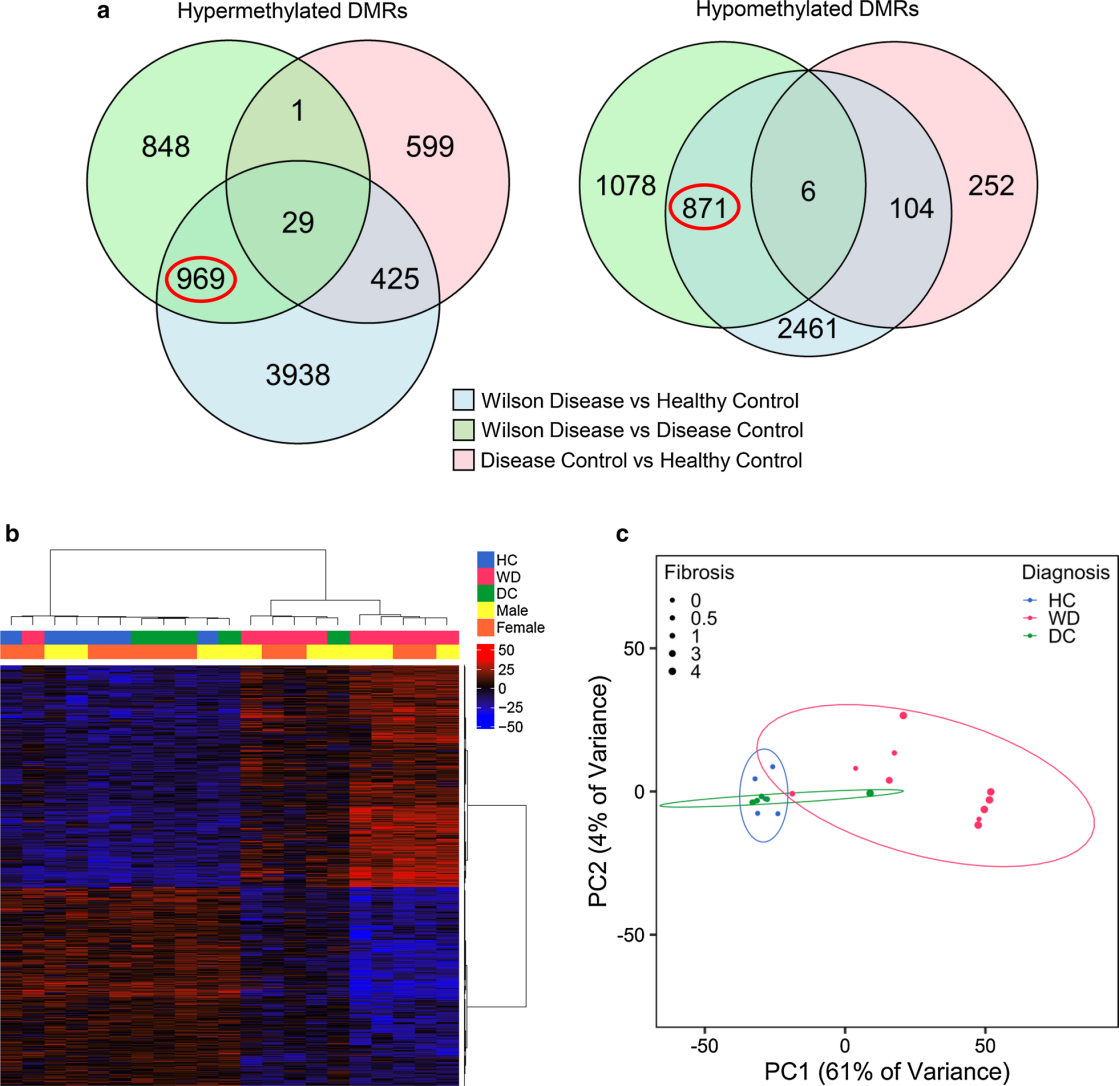
Liver DMRs distinguish patients with WD from controls. **a** WGBS-derived DMRs differentiating WD from both HC and DC, but not DC from HC, were identified (WD *n *= 10, HC *n *= 6, DC *n *= 5). Hypermethylated DMRs have higher methylation in WD, while hypomethylated DMRs have lower methylation in WD. **b** Heatmap of HC, WD, and DC using methylation in WD-specific liver DMRs. Percent methylation for each sample relative to the mean methylation at each DMR is plotted. **c** Principal component analysis using methylation in WD-specific liver DMRs. The size of each point indicates the fibrosis stage of the patient. Ellipses show 95% confidence intervals. For this and all subsequent figures: DMR, differentially methylated region; WD, Wilson disease; WGBS, whole-genome bisulfite sequencing; HC, healthy control; DC, disease control

To identify DNA methylation changes specific to WD in liver, samples from WD (*n *= 10), HC (*n *= 6), and DC (*n *= 5) subjects were analyzed by WGBS for DMRs between each group. 969 hypermethylated and 871 hypomethylated regions were identified that specifically differentiate WD from both HC and DC subjects, but not DC from HC subjects (Fig. 1a). Of these WD-specific DMRs, 18 reached genome-wide significance by FWER (Table 1). Genes near these 18 DMRs included those with known functions in liver development (*BST1*, *FOXA1,* and *VTN*) and transcriptional regulation (*FOXA1*, *MAFB*, *MN1*, *NACC2*, *ZNF689*, and *ZNF785*). Gene ontology analysis of WD-specific liver DMRs showed that genes near hypermethylated DMRs were enriched in functions related to acute inflammatory response, lipid catabolism, and folic acid metabolism (Additional file 1: Fig. S1). In contrast, genes near hypomethylated DMRs were enriched in functions related to humoral immune response, fatty acid transport, and regulation of glycolysis.

**Table 1:** WD-specific liver DMRs with genome-wide significance and associated genes

Chr	Start	End	CpGs	Methylation difference (%)	FWER	Gene	Distance to TSS (kb)	Position
chr21	31557558	31560049	156	− 37	< 0.001	*TIAM1*	0.0	TSS
						*LOC150051*	0.0	TSS
chr12	3199472	3201083	96	− 35	0.004	*TSPAN9*	122.1	Intron
						*PRMT8*	− 180.3	Upstream
chr9	136050881	136052939	57	− 35	0.008	*NACC2*	42.3	Intron
						*UBAC1*	− 89.5	Upstream
chr22	27797270	27798795	87	27	0.018	*MN1*	2.7	Exon
						*LOC100507657*	486.6	Downstream
chr15	99105435	99106614	93	− 13	0.020	*SYNM*	0.4	Exon
						*LRRC28*	− 144.7	Upstream
chr18	22171324	22172395	66	− 27	0.023	*GATA6*-*AS1*	− 2.4	Upstream
chr21	44456208	44457837	56	29	0.023	*LRRC3*	0.7	Exon
						*LRRC3*-*AS1*	− 0.9	Upstream
chr20	40691316	40693007	60	− 28	0.025	*MAFB*	− 2.1	Upstream
chr9	137461602	137462399	37	− 28	0.028	*NSMF*	− 2.3	Upstream
chr9	136418051	136418847	29	32	0.031	*SDCCAG3*	− 7.4	Upstream
						*INPP5E*	21.0	Downstream
chr11	66855700	66857160	56	− 32	0.032	*LRFN4*	− 0.2	Upstream
chr22	39994424	39995559	72	− 24	0.035	*FAM83F*	0.0	TSS
chr4	15702770	15703332	32	− 28	0.038	*BST1*	0.0	TSS
chr14	37597362	37599120	65	− 17	0.038	*FOXA1*	− 2.2	Upstream
chr16	30604376	30605260	48	29	0.043	*ZNF689*	5.5	Exon
						*ZNF785*	− 18.6	Upstream
chr17	28371994	28372583	59	− 20	0.044	*SARM1*	0.3	Exon
						*VTN*	− 1.6	Upstream
chr9	121772727	121773248	32	23	0.048	*DAB2IP*	205.6	Exon
						*TTLL11*	320.4	Downstream
chr5	10649279	10650004	64	18	0.049	*ANKRD33B*	85.0	Exon
						*DAP*	111.3	Downstream

FWER, family-wise error rate

The majority of WD subjects could be clearly distinguished from HC and DC subjects on the basis of methylation levels within WD-specific liver DMRs (Fig. 1b, c). Methylation in these DMRs was not associated with sex, age, BMI, inflammation, or steatosis (Additional file 1: Fig. S2, Additional file 2: Table S3). In contrast, methylation in WD-specific DMRs overall, represented by the first principal component, was associated with fibrosis (*p* = 2.5E−4), which can also be observed in the heterogeneity within WD samples (Fig. 1c). Individually, 23 DMRs (1% of WD-specific DMRs) were associated with fibrosis (Bonferroni-adjusted *p* < 0.05, Additional file 2: Table S3). Additionally, 63 (3%) WD-specific DMRs overlapped with DMRs previously identified in a methylation study of fibrosis in NAFLD subjects [16]. The majority of individual WD-specific DMRs, however, were not significantly associated with fibrosis.

To investigate WD-specific DNA methylation changes in liver prior to onset of fibrosis, early-stage (stage 0–1) patients with WD (*n *= 3) were compared to HC (*n *= 6) and early-stage DC (*n *= 4) subjects. 124 hypermethylated and 70 hypomethylated regions were identified distinguishing early-stage WD patients, which were not associated with demographic covariates (Additional file 1: Fig. S3, Additional file 2: Table S4). 76 of these early-stage regions and 167 associated genes overlapped with WD-specific liver DMRs identified from all WD patients, a significant enrichment (Hyper: *p* = 5.2E−82, Hypo: *p* = 1.4E−31). Furthermore, in the tx-j mouse model of WD, 10 genes selected based on human WD differential methylation were significantly differentially expressed compared to control in liver at an early stage of pathology (*q* < 0.05, Additional file 1: Table S5). Of these genes, *Gata6*, *Hdac5*, *Pmpca*, *Pnpla7*, and *Tspan9* were upregulated, while *Foxa1*, *Mafb*, *Nacc2*, *Pcx*, and *Vtn* were downregulated. Together these results suggest that methylation differences detected in human WD liver have functional consequences and are related to early pathogenesis mechanisms rather than a result of late-stage fibrosis.

### WD-specific hypermethylated liver DMRs are enriched in liver enhancers and flanking active liver promoters

**Fig. 2:**
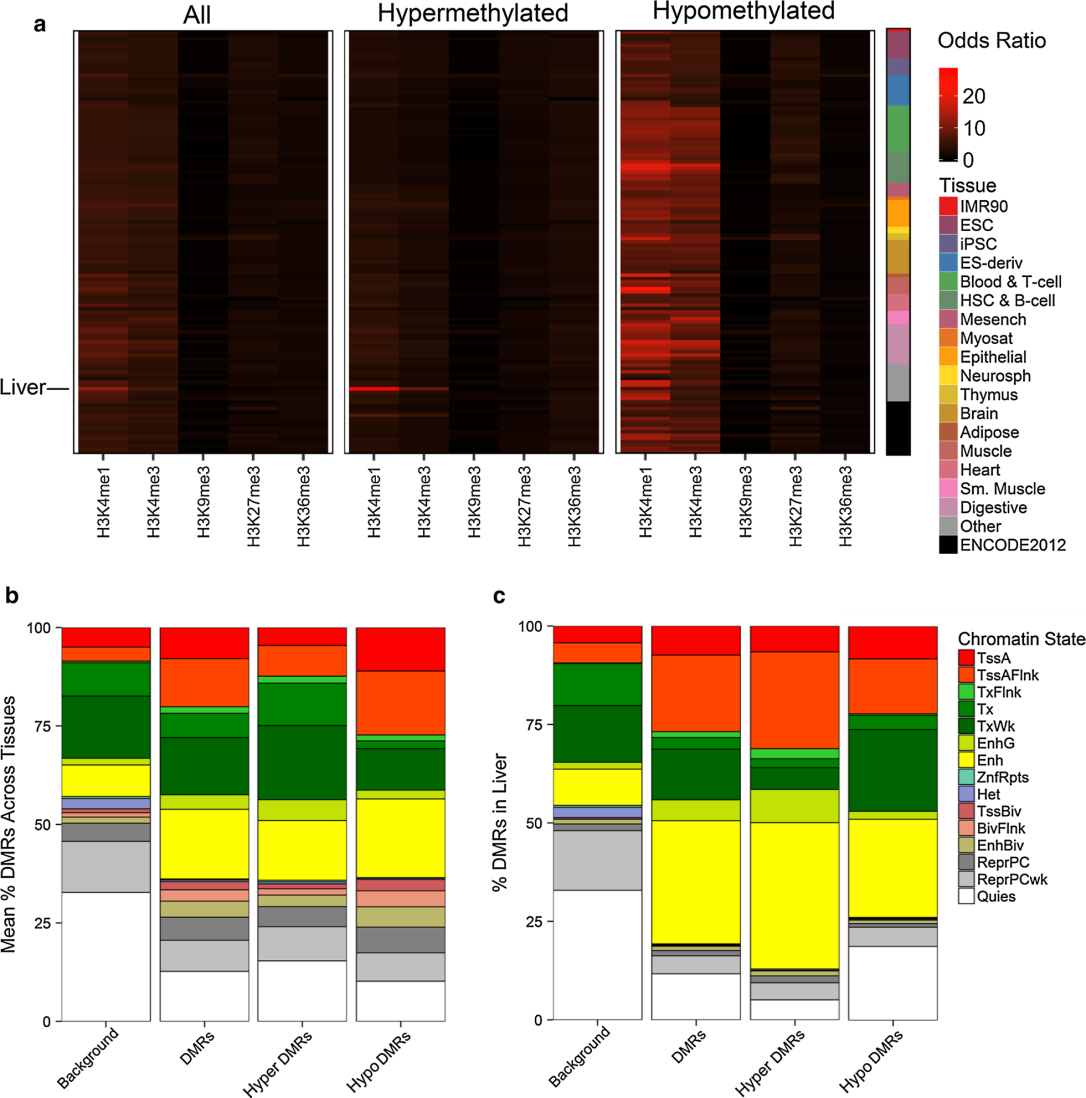
WD-specific hypermethylated liver DMRs are enriched in liver enhancers and flanking active liver promoters. **a** WD liver DMRs were overlapped with histone modification ChIP-seq peaks from the Epigenome Roadmap using LOLA and the odds ratio was plotted for all tissues. **b**, **c** WD liver DMRs were overlapped with chromatin states from the Epigenome Roadmap using LOLA and the percent of DMRs and background regions overlapping each state was plotted as **b** the mean overlap for all tissues or **c** overlap for liver

To functionally annotate the WD-specific liver DMRs identified from all samples, histone modification ChIP-seq peaks and chromatin state predictions from 127 cell and tissue types in the Roadmap Epigenomics Project [17] were compared to DMR chromosomal locations for enrichment (Fig. 2). Hypermethylated WD-specific DMRs were highly significantly enriched for liver H3K4me1 and H3K4me3 histone modification marks overlapping 87% and 50% of hypermethylated DMRs, respectively (H3K4me1 odds ratio = 28.6, false discovery rate (FDR) *q* < 1.0E−319; H3K4me3 odds ratio = 8.8, FDR *q* = 7.6E−210). In contrast, hypomethylated WD-specific DMRs were enriched in H3K4me1 and H3K4me3 marks across many tissue types. WD-specific liver DMRs were next overlapped with ChromHMM chromatin state predictions, which use histone modification ChIP-seq data to segment the genome into 15 functional states. Combined WD-specific DMRs showed significant enrichment in enhancers (Enh, yellow) and regions flanking active transcription start sites (TssAFlnk, orange) compared to background across all tissues (Fig. 2b), with a markedly pronounced enrichment of these functional regions (Fig. 2c). Hypermethylated WD-specific DMRs individually were also specifically enriched in liver enhancers and regions flanking active transcription start sites (Additional file 1: Fig. S4; Enh odds ratio = 11.6, FDR *q* = 5.5E−278; TssAFlnk odds ratio = 10.1, FDR *q* = 3.7E−192). Regions flanking transcribed regions were also significantly enriched, but only made up a small portion of hypermethylated DMRs.

### WD-specific hypermethylated DMRs in liver are enriched in liver-associated transcription factor binding sites

**Fig. 3:**
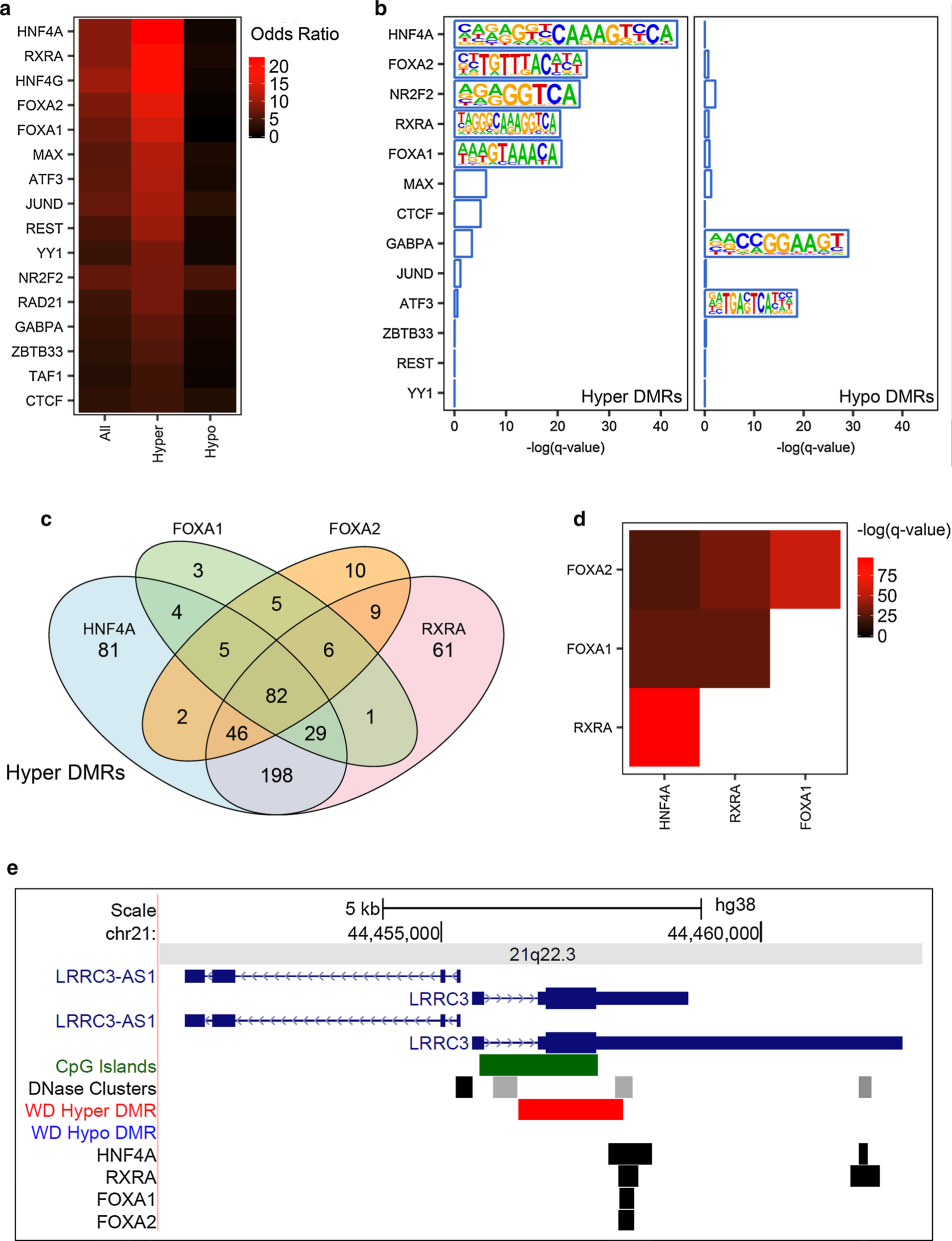
WD-specific hypermethylated liver DMRs are enriched in liver-associated transcription factor binding sites. **a** WD liver DMRs were overlapped with available liver transcription factor ChIP-seq peaks from ENCODE using LOLA and the odds ratio was plotted and sorted by hypermethylated DMR odds ratio. **b** WD liver DMR sequences were tested for enriched known transcription factor motifs using HOMER and factors with liver ChIP-seq data were plotted. **c** Hypermethylated WD liver DMRs overlapping with top liver transcription factor ChIP-seq peaks were overlapped. **d** Transcription factor overlap enrichment. **e** UCSC Genome Browser view of overlapping transcription factor binding sites and a hypermethylated DMR at *LRRC3*

To determine the potential association of methylation differences at WD-specific liver DMRs with transcription factor binding, DMRs were overlapped with transcription factor ChIP-seq peaks in liver from ENCODE (Fig. 3a) and assessed for enrichment of known transcription factor binding site sequence motifs (Fig. 3b). Hypermethylated DMRs were enriched in liver binding sites and sequence motifs for HNF4A, RXRA, FOXA1, and FOXA2, while hypomethylated DMRs had no enrichment for these factors (Additional file 1: Fig. S5). When hypermethylated DMRs overlapping binding sites for HNF4A, RXRA, FOXA1, and FOXA2 were compared, they were enriched for overlapping the same DMRs, including 82 regions containing all four of these factors (Fig. 3c, d). One such DMR was at *LRRC3*, which was hypermethylated specifically in WD liver with genome-wide significance, and contains overlapping liver binding sites for all four of these factors (Fig. 3e).

### WD progression-associated liver DMRs reveal dysregulation of complement activation and embryonic development pathways with increasing disease severity

**Fig. 4:**
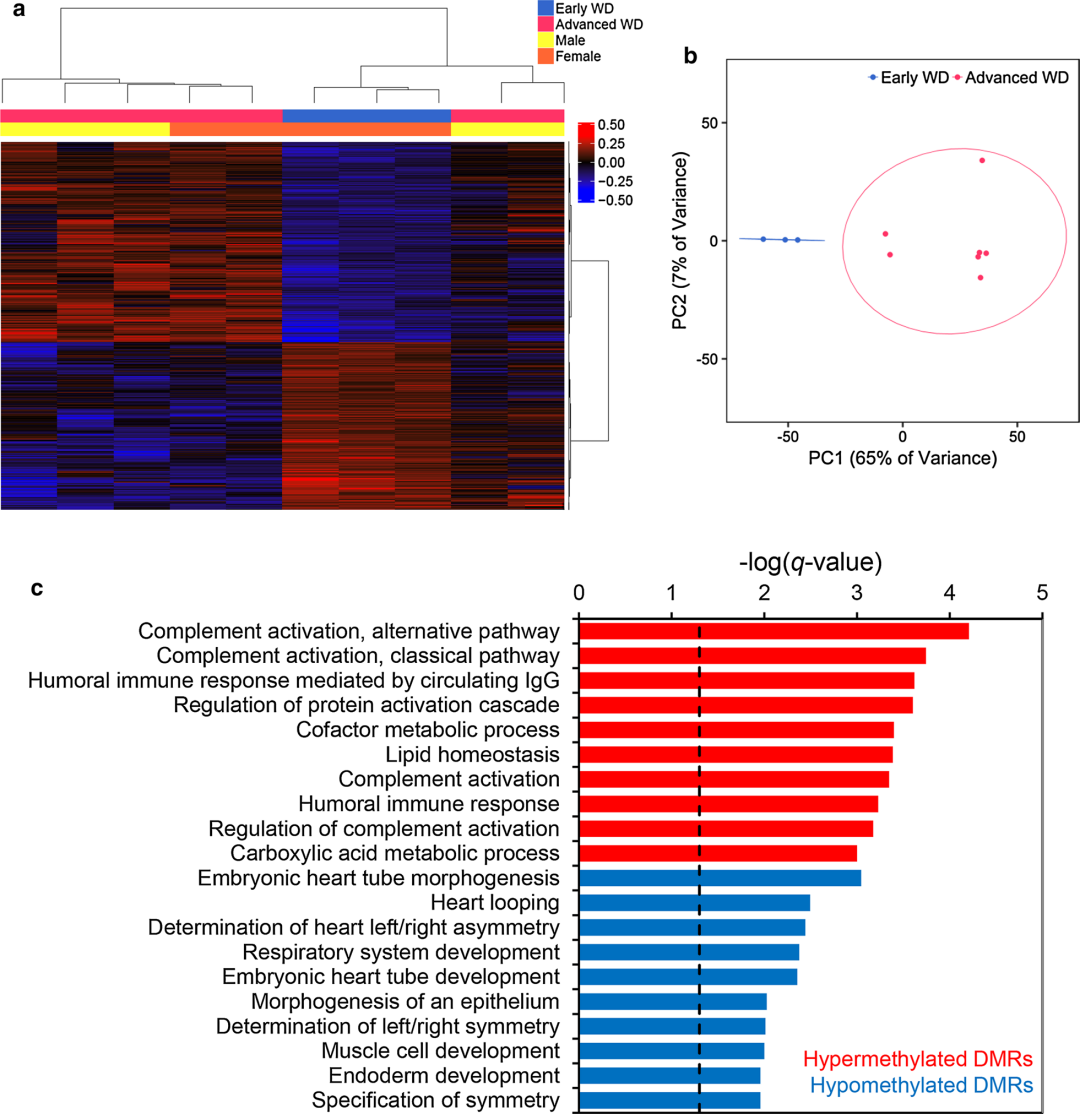
Liver DMRs differentiating early- from advanced-stage patients with WD. WD progression-associated liver DMRs are enriched for genes involved in complement activation and embryonic development. Hypermethylated DMRs have higher methylation in advanced WD, while hypomethylated DMRs have lower methylation in advanced WD (early WD *n *= 3, advanced WD *n *= 7). **a** Heatmap using methylation in early versus advanced WD liver DMRs. Percent methylation for each sample relative to the mean methylation at each DMR is plotted. **b** Principal component analysis using methylation in early versus advanced WD liver DMRs. Ellipses show 95% confidence intervals. **c** Results from GREAT functional enrichment analysis of early versus advanced WD liver DMRs compared to background. Top 10 terms from gene ontology databases for hypermethylated or hypomethylated DMRs with FDR *q* < 0.05 are shown (dotted line indicates the significance threshold). For this and all subsequent figures: FDR; false discovery rate

To determine the DNA methylation changes in liver associated with progression of WD, early (stage 0–1) and advanced fibrosis stage (≥ 2) WD patients were compared and assessed for DMRs (early WD *n *= 3, advanced WD *n *= 7). 1339 hypermethylated and 1119 hypomethylated regions were identified as changing with disease progression, and subjects could be separated by stage on the basis of methylation in these DMRs (Fig. 4a, b). However, methylation was not associated with covariates (Additional file 2: Table S6). Among these WD progression DMRs, 15 reached genome-wide significance (Additional file 1: Table S7). Genes near these significant DMRs included those involved in blood coagulation (*F7*, *F10*), immune activation (*RNF123, SIGLEC15*) and mitochondrial function (*KAT2A*, *LIAS*, and *SARDH*). Functional enrichment analysis of all WD progression DMRs revealed that hypermethylated regions were enriched for genes involved in complement activation, humoral immune response, and lipid homeostasis, while hypomethylated regions were enriched for genes involved in heart, respiratory, muscle, and endoderm development (Fig. 4c). These data suggest that DNA methylation alterations of genes associated with complement activation and embryonic development occur during WD progression.

### WD-associated liver DMR genes are enriched in drug targets

Identification of genes with differential methylation in liver that are associated with WD diagnosis and severity offers the opportunity to predict therapeutics that may interact with these genes and potentially alter WD pathology. To identify drugs associated with WD DMRs, known drug–gene interactions were obtained from the Drug Gene Interaction database [18] and overlapped with WD DMR genes (Additional file 2: Table S8). The most significantly enriched drug was bepridil, which is a calcium antagonist known to affect mitochondrial function [19] that interacts with 4 genes with differential methylation in early-stage WD: *ATP1A1*, *CACNA1H*, *TNNC1*, and *VIPR2* (*p* = 1.2E−4). WD-specific liver DMR genes were significantly enriched for interactions with melphalan, which has been used to treat liver metastases [20] and is associated with the *FANCC*, *GSTP1*, *MGMT*, *OPLAH*, and *PLAT* genes (*p* = 6.5E−4). Although the effects of these drugs on WD pathology cannot be predicted based on this analysis, these data do suggest compounds for further investigation in WD model systems.

### WD-associated liver methylation is not influenced by cell-type changes

DNA methylation changes observed in a bulk tissue such as liver can be affected by differences in proportions of individual cell types between samples. To determine whether cell-type changes influenced DMRs identified in this study, we examined sets of promoters specifically hypermethylated in human hepatocytes (HEP), hepatic stellate cells (HSC), or liver sinusoidal epithelial cells (LSEC) [21]. Out of all of the identified WD DMRs, only 63 WD-specific, 3 early-stage WD-specific, and 86 WD progression DMRs overlapped with cell-type-specific methylated promoters (Additional file 1: Fig. S6a). Less than 5% of DMRs from each comparison were located at a cell-type-specific methylated promoter. Percent methylation at cell-type-specific promoters was compared between all sample groups used to identify DMRs (Additional file 1: Fig. S6b-d, Additional file 2: Table S9). The majority of cell-type-specific promoters were not differentially methylated between any of the sample groups. Compared to HC, WD samples were nominally differentially methylated at 7% of HEP promoters, 4% of HSC promoters, and 7% of LSEC promoters (*p* < 0.05). The largest effect was observed in advanced compared to early-stage WD, where 8% of HEP promoters, 9% of HSC promoters, and 13% of LSEC promoters were nominally differentially methylated (*p* < 0.05). These results suggest that alterations in cell-type proportions do not have a major effect on differential methylation observed in WD liver.

### DMRs in blood distinguish WD from controls

**Fig. 5:**
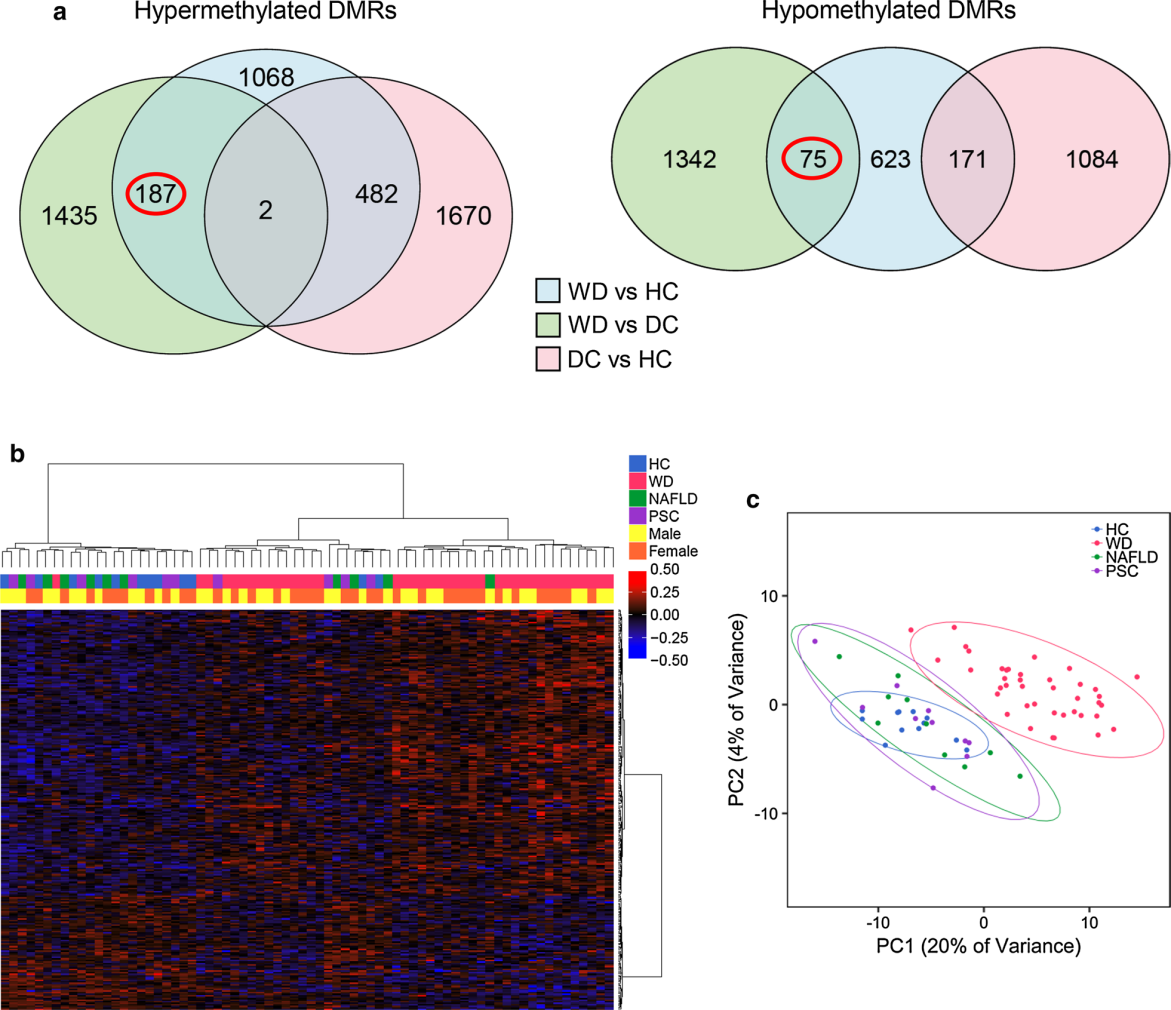
Blood DMRs distinguish patients with WD from controls. **a** WGBS-derived DMRs differentiating WD from both HC and DC, but not DC from HC, were identified (WD *n *= 40, HC *n *= 12, DC *n *= 20). **b** Heatmap of HC, WD, NAFLD, and PSC blood using methylation in WD-specific blood DMRs. Percent methylation for each sample relative to the mean methylation at each DMR is plotted. **c** Principal component analysis using methylation in WD-specific blood DMRs. Ellipses show 95% confidence intervals

While the identification of liver WD-specific DMRs is most relevant for disease insights, we asked whether a similar epigenomic signature could be identified in more accessible blood samples as potential WD biomarkers. A total of 82 whole blood samples in two independent cohorts were available for DNA extraction and methylome analysis (Additional file 1: Table S10, Additional file 2: Table S11). Patients with WD were all recruited at the time of diagnosis and not on anti-copper treatment. The stage of fibrosis was available, as derived directly from liver biopsy or from non-invasive assessment, for NAFLD and PSC subjects, ranging from no fibrosis to cirrhosis. Whole blood methylomes from WD (*n *= 40), HC (*n *= 12), and DC (which includes NAFLD and PSC patients, *n *= 20) were assessed by WGBS, and DMRs were called for each pairwise comparison (Fig. 5a). 187 hypermethylated and 75 hypomethylated WD-specific DMRs were identified distinguishing WD from both HC and DC patients. Using methylation levels in these WD-specific blood DMRs, the majority of patients with WD separated from HC and DC patients (Fig. 5b, c). In contrast, methylation in the majority of DMRs was not associated with demographic covariates (Additional file 2: Table S12).

Due to the enrichment of liver WD-specific DMRs in liver enhancers, chromatin state enrichment in blood WD-specific DMRs was also assessed (Additional file 1: Fig. S7). Overall, DMRs were enriched for immune cell enhancer regions, and especially those predicted in hematopoietic stem cells, which overlapped 51% of DMRs (odds ratio = 9.1, FDR *q* = 6.1E−58). Additionally, hypermethylated regions were enriched more in monocyte, neutrophil, B cell, and natural killer cell enhancers, while hypomethylated regions were enriched more in T cell enhancers and promoters.

### DNA methylation in blood reflects a subset of WD-specific liver DMRs

The number of WD-specific DMRs identified in blood was much lower than those identified in liver, as expected. However, 99 (22%) of the genes near blood DMRs overlapped with those identified in liver, a significant enrichment (Hyper: *p* = 1.1E−25, Hypo: *p* = 2.0E−6, Additional file 1: Fig. S8a). Genes near the 10 DMRs with overlapping locations and common direction in blood and liver included *CSAD*, *ITGB7*, *LSM12*, *PSMD9*, *RRN3P2*, *SNX29P2*, and *PFN3*. In particular, a liver DMR in an intron of *HDAC5* and upstream of *LSM12* was specifically and significantly hypermethylated in WD liver and blood (Additional file 1: Fig. S8b–d).

### Human and mouse WD DMR genes overlap within and across tissues

To determine whether DMR genes associated with *ATP7B* loss of function are conserved between humans and mice, human DMR genes in liver and blood were overlapped with DMR genes previously identified in fetal liver of the WD model tx-j mice compared to wild-type C3H mice (Additional file 1: Fig. S9, Additional file 2: Table S13) [15]. Twenty genes were differentially methylated in the same direction in both human liver and mouse fetal liver (hyper: *q* = 7.7E−2, hypo: *q* = 4.7E−4). Among the genes with conserved differential methylation upon *ATP7B* loss of function were *ALKBH5*, *GRID2IP*, *KDELR2*, *CACNA1H*, *CAMK2B*, and *WNT11*. In blood, 7 genes were differentially methylated in the same direction as mouse fetal liver (hyper: *q* = 6.6E−3, hypo: *q* = 1.3E−1). Of these, *ZNF750* and *MAD1L1* were also differentially methylated in human liver. The conserved differential methylation of these genes suggests they represent important early perturbations in WD pathology.

### DMRs in blood distinguish patients with hepatic and neurologic WD phenotype

**Fig. 6:**
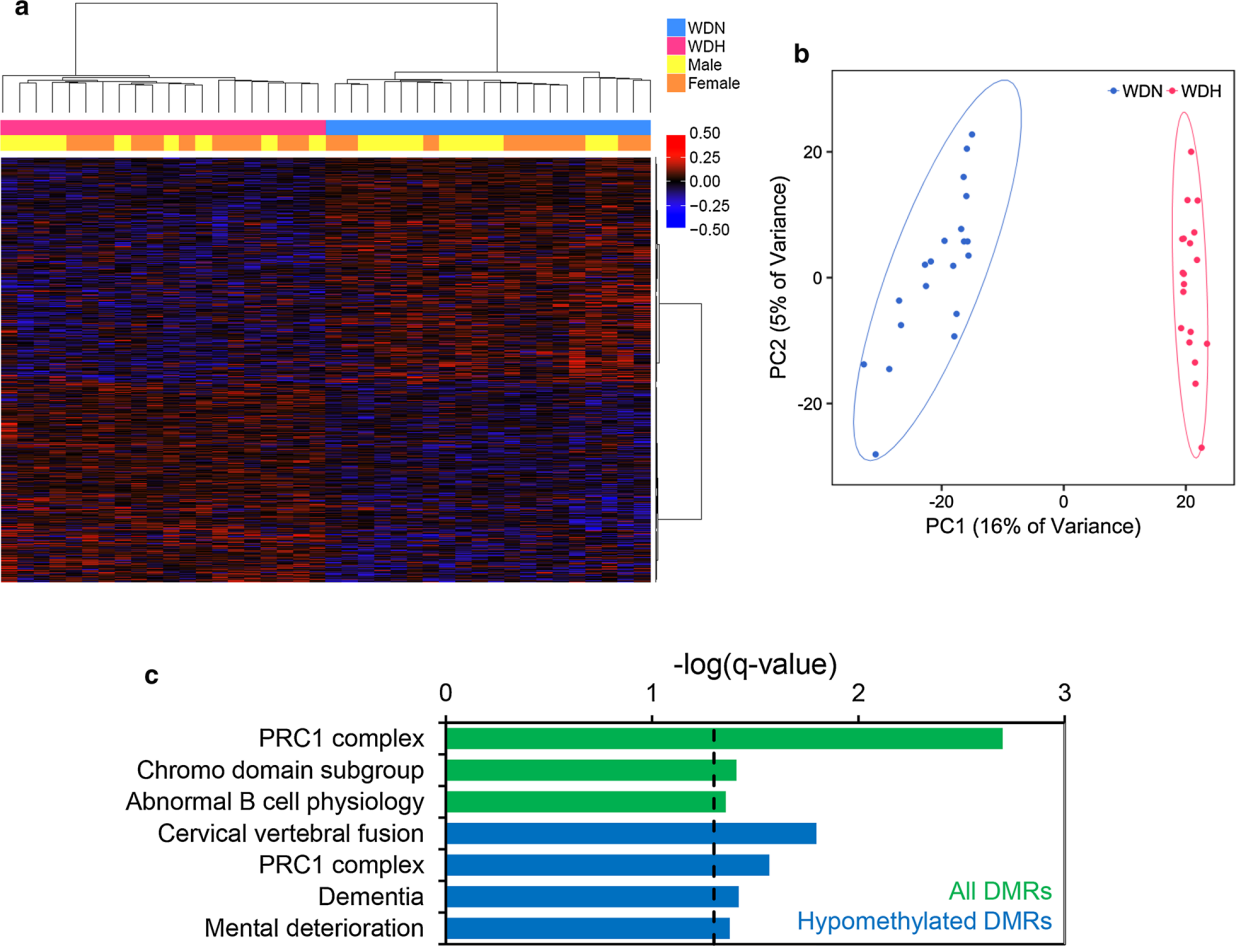
Blood DMRs distinguish patients with hepatic (H) or neurologic (N) WD. DMRs differentiating WDH from WDN were identified. **a** Heatmap using methylation in WDH versus WDN blood DMRs. Percent methylation for each sample relative to the mean methylation at each DMR is plotted (WDH *n *= 20, WDN *n *= 20). **b** Principal component analysis using methylation in WDH versus WDN blood DMRs. Ellipses show 95% confidence intervals. **c** Results from GREAT functional enrichment analysis of WDH versus WDN blood DMRs compared to background. Terms from gene ontology databases with FDR *q* < 0.05 are shown, with a dotted line indicating the significance threshold. “All DMRs” (green) refers to both hypermethylated and hypomethylated DMRs; “Hypomethylated DMRs” (blue) refers to DMRs with lower methylation in WDH compared to WDN

Phenotypic variation is an important feature of WD that is not explained by genotype, so we analyzed methylation in blood to determine whether DMRs were present that differentiated WD patients with hepatic (WDH) and neurologic (WDN) symptoms (WDH *n *= 20, WDN *n *= 20). A total of 1346 regions were hypermethylated, while 1514 regions were hypomethylated in WD patients with hepatic symptoms compared to those with neurologic symptoms (Fig. 6a, b). Patients could clearly be clustered by phenotype, suggesting epigenetic differences exist in the blood that are associated with WD symptoms. In contrast, methylation in WD phenotype DMRs was not associated with covariates (Additional file 2: Table S14). Genes near all DMRs were enriched for functions in abnormal B cell physiology, while those near regions hypomethylated in WDH were enriched for functions in dementia, mental deterioration, and for belonging to the chromatin-modifying polycomb repressive complex 1 (PRC1) (Fig. 6c). Resembling the WD-specific blood DMRs, WD phenotype DMRs were enriched in immune cell enhancers, and especially those in hematopoietic stem cells (Additional file 1: Fig. S7c, d). Uniquely, WD phenotype DMRs were enriched in bivalent enhancers, which are defined by both H3K4me1 and H3K27me3 and regulated by PRC1, and were especially enriched in T cells (odds ratio = 4.6, FDR *q* = 1.5E−171).

### WD phenotype blood DMRs classify hepatic and neurologic WD in independent cohort

**Fig. 7:**
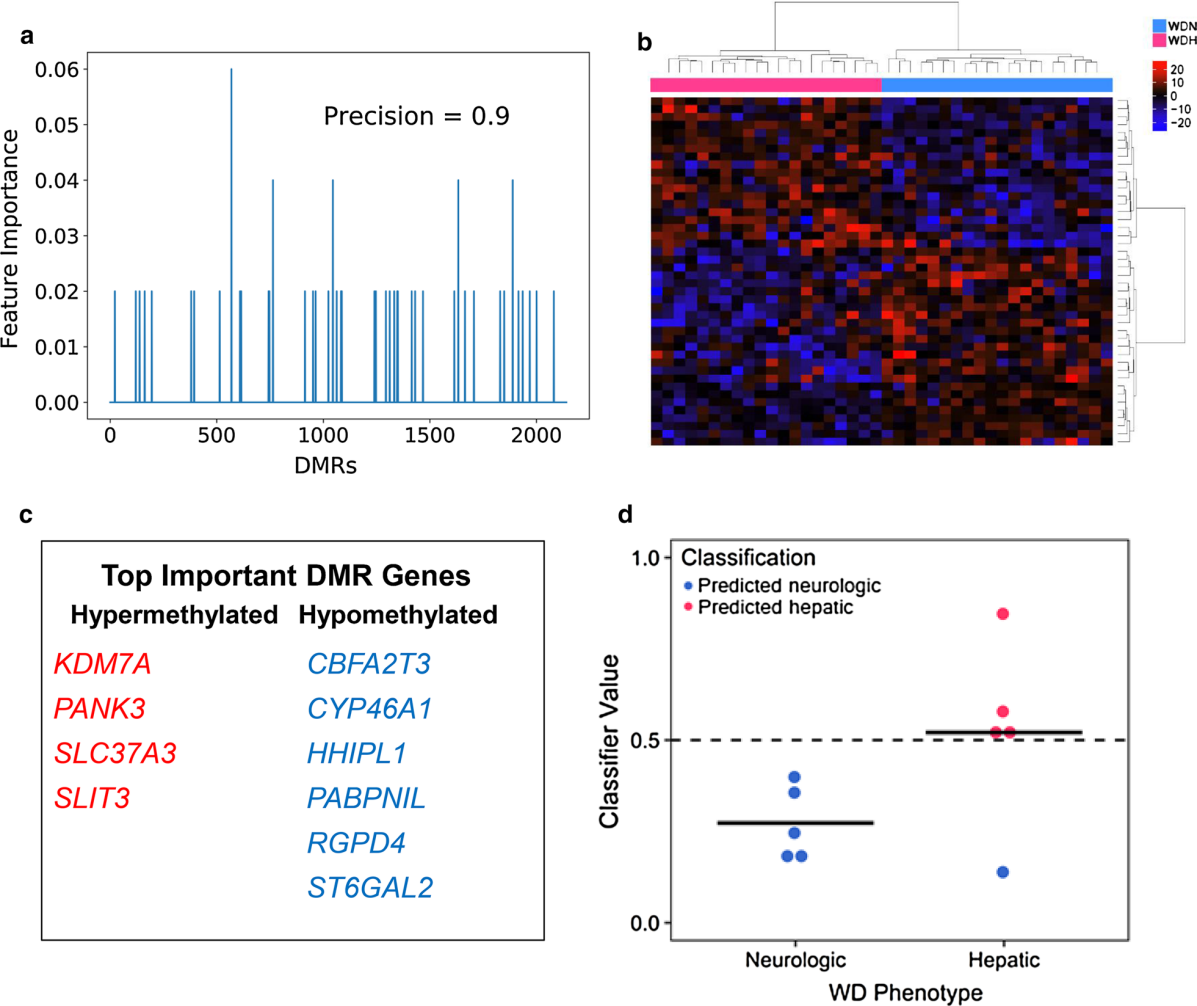
Selected WD phenotype blood DMRs classify hepatic from neurologic WD in an independent test cohort. A classifier using the AdaBoost algorithm was trained with methylation values at WD phenotype DMRs in WD patient blood (WDH *n *= 20, WDN *n *= 20). **a** Feature importance was determined for each of the 2860 DMRs after training. **b** Heatmap of 44 DMRs used in classifier using methylation in training samples. **c** Genes near DMRs with feature importance ≥ 0.04. **d** Classification results in test cohort of WD patients. In the plot, the column represents true phenotype, while the color represents predicted phenotype (WDH *n *= 5, WDN *n *= 5, precision = 0.9, ROC AUC = 0.8)

Distinguishing between WD phenotypes using blood-based biomarkers has a potential application in patient diagnostics. To assess the accuracy of the identified WD phenotype blood DMRs to classify WD patients as either the hepatic or neurologic form, an Adaboost classifier was trained on blood methylation in these regions in the original patient cohort and tested on an independent cohort (WDH *n *= 5, WDN *n *= 5). 44 DMRs were identified with feature importance greater than zero and were used in the model (Fig. 7a, b). Genes near these DMRs included several with known neurologic function, such as *CHRNB3*, *CYP46A1, GALR1, LHX4, NGF*, *NPTXR*, *SHH*, and WNT7B, and hepatic function, including *ONECUT1* (Fig. 7c, Additional file 2: Table S15). Importantly, the classifier was able to correctly identify all but one of the independent test samples as having either the hepatic or neurologic form of WD (precision = 0.9, receiver operating characteristic (ROC) area under the curve (AUC) = 0.8, Fig. 7d). These results suggest that WD phenotype can be identified in patients using blood DNA methylation in these 44 regions.

## Discussion

This is the first study to investigate methylation changes in patients with WD at a genome-wide level through WGBS. This epigenomic signature reveals methylation changes occurring over liver-specific enhancers and transcription factor binding sites, demonstrating that WD is a genetic disease whose progression and, potentially, pathogenesis is associated with epigenetic changes. We demonstrate a specific liver pathogenesis-related DNA methylation signature in patients with WD compared to healthy subjects and subjects with other liver diseases. A subset of these DNA methylation differences were identified both in the liver and in the blood from distinct cohorts of patients, supporting the premise that methylation markers in the blood can be reflective of disease pathogenesis in another tissue. Furthermore, DNA methylation differences also distinguished patients on the basis of hepatic versus neurologic presentation, specifically at genes encoding epigenetic factors with mechanistic significance, such as those in PRC1. A small subset of these regions could classify WD patient phenotypes in an independent cohort, demonstrating the diagnostic potential of DNA methylation. This epigenomic signature of WD reveals insights into gene pathways, gene–drug interactions, and potential biomarkers that may be clinically useful in early intervention and treatment of WD.

Our previous data in the tx-j mouse model of WD demonstrated a global reduction of hepatic cell growth and maturation deficiency in fetal livers, alleviated by maternal dietary supplementation with the methyl donor, choline [22]. Differential methylation was also observed in fetal liver of tx-j mice, which was ameliorated by choline supplementation [15]. Differentially methylated genes identified in patient livers significantly overlapped with those in tx-j fetal liver and included several genes encoding transcription factors important in liver development. *GATA6* is involved in multiple stages of liver maturation and highly expressed in hepatocytes during early fetal development [23]. *FOXA1* is crucial in initiating specification during liver embryogenesis [24] and is also expressed in adult livers, whereas *VTN*, a FOXA1 target gene, is involved in embryonic stem cell maturation [25] and has been shown to be upregulated in fetal hepatic stellate cells [26]. *Gata6*, *Foxa1*, and *Vtn* were also found to be differentially expressed in adult tx-j liver. These DNA methylation signatures of WD point to liver development and regeneration pathways which could be in turn affected by methyl donor dietary factors both in the fetal and adult life.

Hypermethylated DMRs identified in WD were specifically enriched in liver-specific enhancers and transcription factor binding sites, which is a novel finding for the WD field. Hypermethylated DMRs in liver-specific enhancers may reflect specific deficits in enhancer usage or HNF4A binding. HNF4A is a crucial transcription factor in hepatocyte maturation, necessary to maintain the epithelial phenotype of hepatocytes [27]. A previous report showed impaired binding of HNF4A and RXR to promoter response elements in patients with WD [28]. In addition, dysregulation of liver X receptor/retinoid X receptor was implicated in the pathogenesis of WD [29]. Liver binding sites for HNF4A, RXR, FOXA1, and FOXA2 were found to overlap at a significantly hypermethylated WD-specific liver DMR in the promoter of *LRRC3*. Another DMR at *LRRC3* was significantly hypermethylated in advanced compared to early-stage WD. Previously, *LRRC3* has been associated with susceptibility to platinum-induced liver injury and oxidative stress [30]. Our results demonstrating hypermethylation over HNF4A and other transcription factor binding sites within liver-specific enhancers and promoters provides a new potential explanation for these prior molecular findings in WD pathogenesis.

In addition, the insights gained from this epigenomic study of WD may shed light on the controversial relationship between copper accumulation and potentially reduced risk of liver cancer in WD [31, 32]. Among the most striking findings were that the DMR genes overlapping between liver and blood have major roles in epigenetic mechanisms in cancer. In particular, HDAC5 activation is implicated with hepatocyte proliferation and hepatocarcinogenesis [33] and has been involved in lipid metabolism and fatty acid oxidation [34]. Both *NCOR2* and *CTBP2* were hypermethylated in liver and blood of WD patients and function as transcriptional co-repressors that are dysregulated in cancer. *NCOR2* interacts with both nuclear receptors, including LXR and RXR, and class IIa HDACs, such as HDAC5 [35, 36]. *NCOR2* is also differentially expressed in bladder, breast, and prostate cancers [35]. *CTBP2* functions to recruit PRC2 proteins to add H3K27me3 during differentiation and also interacts with HDACs [37]. Further, *CTBP2* is overexpressed in prostate cancer and associated with tumor progression [38]. Other genes, including *GATA2*, *GADD45B*, and *MIR126*, are differentially methylated in WD liver and blood, also play a role in epigenetic mechanisms in cancer. The dysregulation of these epigenetic regulators in WD may impact the susceptibility of these patients to cancer.

Several prior studies have attempted to identify specific DNA methylation changes in whole blood or peripheral blood mononuclear cells (PBMCs) as indicators of liver disease. In particular, analyses of patients with NASH identified DNA methylation changes over genes associated with collagen content [11] and steatosis [39]. Our in-depth analysis identified DMRs that could differentiate early- from advanced-stage WD and hepatic from neurologic phenotypes at the time of diagnosis. To our knowledge, this is the first study identifying a combination of pathogenesis-meaningful markers with the potential to contribute to the diagnosis in association with clinical and genetic parameters. In our analysis of advanced compared to early WD liver samples, we identified hypermethylated genes involved in the complement pathway, which were previously shown to be serum biomarkers of WD [40]. Our study describes a set of differentially methylated genes from whole blood overlapping with those identified in WD liver, suggesting that these loci could be pursued as potential blood biomarkers of WD at various stages of disease progression in future studies.

Limitations of this study include the use of whole blood and whole liver tissue, as both are mixtures of many different cell types. The influence of cell type was assessed in liver, where it was found that the large majority of promoters hypermethylated in specific liver cell types were not associated with differential methylation in WD liver. A second limitation was the lack of liver controls from individuals of a healthy weight. This was controlled for by examining BMI, which was not associated with DMR methylation. The changes in DNA methylation of genes in pathways related to fatty acid oxidation and lipid catabolism are consistent with prior evidence of downregulated lipid metabolism pathways in WD animal models [8]. A third limitation was the relatively low coverage of the methylation analysis which may limit the ability to detect all possible methylation differences. However, the relatively large number of samples allowed us to capture a large number of DMRs that were validated in independent cohorts providing the proof of principle of a role for DNA methylation in WD. In addition, the significant costs of the described sequencing methodologies could be seen as limiting factor to the future applicability of DMR markers as diagnostic tests for WD. Using reduced representation of specific WD predictive DMRs will significantly reduce the costs, increase the sequencing coverage, and make this an accessible technology for clinical practice.

## Conclusions

WD represents a very distinct genetic condition where mutations in *ATP7B* interact with copper, diet, and metabolism, affecting its complex and varied phenotype, so epigenetic indications of disease progression in blood or other accessible tissue would clearly be clinically useful. These DMRs likely represent an epigenomic signature of WD that includes direct alterations from increased copper levels, responses to disease progression, and inputs from the environment such as diet. In addition, the identification of DNA methylation changes over genes encoding drug-targetable epigenetic modifiers, such as *HDAC5*, may reveal insights into repurposing of existing medications in WD treatment.

## Methods

### Human liver biopsies

Samples from patients with WD were obtained from the University of Heidelberg (Heidelberg, Germany) and the California Pacific Medical Center, Ibrahim El-Hefni Biorepository (San Francisco, California, USA). The liver samples from University of Heidelberg were derived from explanted livers of patients with WD who underwent liver transplant. Liver samples from California Pacific Medical Center were obtained through percutaneous liver biopsies performed for diagnostic and staging purposes. Liver samples from DC and HC subjects were obtained from the California Pacific Medical Center and the University of California, Davis GI biobank (Additional file 1: Table S1, Additional file 2: Table S2). DC percutaneous liver biopsies were taken from patients with NAFLD. HC liver biopsies were obtained at the time of bariatric surgery from subjects without diabetes, with less than 5% steatosis and no inflammation on histology. Biopsies were graded on inflammation, steatosis, and inflammation as previously described [41].

### Human whole blood samples

Samples from patients with WD (different groups than patients providing liver biopsies) were obtained from the Institute of Psychiatry and Neurology (Warsaw, Poland). HC samples were obtained from volunteers in Warsaw and the local Sacramento community. DC samples were obtained from patients with a diagnosis of NAFLD and PSC who presented consecutively for evaluation at UC Davis Hepatology clinic and consented to provide whole blood for DNA extraction to be stored in UC Davis GI Division Biobank. NAFLD and PSC were chosen as DC as both conditions should be considered in the differential diagnosis of WD, NAFLD patients present epigenetic changes, and PSC is associated also with copper accumulation. In the HC and WD groups, whole blood samples were matched by age, sex, and BMI (Additional file 1: Table S10, Additional file 2: Table S11). Patients with other chronic liver diseases (including hepatitis B or C, autoimmune hepatitis, alcoholic liver disease, primary biliary cholangitis, hemochromatosis) were excluded. Patients with WD were recruited at the time of diagnosis, determined by Leipzig criteria, and, accordingly, were not on anti-copper treatment. WD patients in the test cohort were selected to have the *ATP7B* H1069Q missense mutation. Patients were categorized according to their prevalent phenotype as having hepatic or neurologic presentation. All samples were de-identified, shipped on dry ice, and stored at − 80 °C for further analysis.

### Whole-genome bisulfite sequencing (WGBS) and analysis

WGBS libraries were prepared from bisulfite-converted DNA using the TruSeq DNA Methylation kit (Illumina, San Diego, CA, USA). Libraries from liver and blood training samples were sequenced on the HiSeq4000, while libraries from blood test samples were sequenced on the HiSeq2000 (Illumina, San Diego, CA, USA). Reads were aligned to the hg38 reference genome, and DMRs were identified between HC, WD, and DC samples. All code for WGBS data analysis is available on GitHub (https://github.com/cemordaunt/WilsonDiseaseEpigenome).

Additional methods about WGBS data analysis and mouse model studies are presented in Additional file 1: Supplemental methods.

## Additional files

**Additional file 1.** Additional methods, figures, Tables S1, S5, S7, S10, and S16, and references.

**Additional file 2.** Additional Tables S2–S4, S6, S8, S9, and S11–S15.

## Abbreviations

WD: Wilson disease
WGBS: whole-genome bisulfite sequencing
DMR: differentially methylated region
HNF4A: Hepatocyte Nuclear Factor 4 alpha
RXRA: Retinoid X Receptor alpha
FOXA1: Forkhead Box A1
FOXA2: Forkhead Box A2
ATP7B: ATPase copper transporting beta
SAHH: *S*-adenosylhomocysteine hydrolase
SAH: *S*-adenosylhomocysteine
NAFLD: non-alcoholic fatty liver disease
PSC: primary sclerosing cholangitis
HC: healthy control
DC: disease control
FWER: family-wise error rate
FDR: false discovery rate
Enh: enhancers
TssAFlnk: regions flanking active transcription start sites
HEP: hepatocytes
HSC: hepatic stellate cells
LSEC: liver sinusoidal epithelial cells
HDAC5: histone deacetylase 5
WDH: Wilson disease with hepatic symptoms
WDN: Wilson disease with neurologic symptoms
PRC1: polycomb repressive complex 1
ROC: receiver operating characteristic
AUC: area under the curve
PBMC: peripheral blood mononuclear cell
