# Supplementary material for: Epigenomic signatures in liver and blood of Wilson disease patients include hypermethylation of liver-specific enhancers
Source: Epigenetics Chromatin. 2019 Feb 1;12:10. doi: 10.1186/s13072-019-0255-z (PMC6357467; doi:10.1186/s13072-019-0255-z)
Supplement: Supplementary file 1 — Additional file 1. Additional methods, figures, Tables S1, S5, S7, S10, and S16, and references. [file 13072_2019_255_MOESM1_ESM.docx]

**Additional Material**

**Epigenomic signatures in liver and blood of Wilson disease patients include hypermethylation of liver-specific enhancers**

Charles E. Mordaunt, Dorothy A. Kieffer, Noreene M. Shibata, Anna Członkowska, Tomasz Litwin, Karl-Heinz Weiss, Yihui Zhu, Christopher L. Bowlus, Souvik Sarkar, Stewart Cooper, Yu-Jui Yvonne Wan, Mohamed Ali, Janine M. LaSalle*, and Valentina Medici*

**Table of Contents**

Section Page

Additional Methods 2

Additional Figures 8

Additional Tables 19

Additional References 24

**Additional Methods**

*Human liver and whole blood whole-genome bisulfite sequencing (WGBS) and analysis*

Sample processing: DNA from liver and whole blood was isolated using the QIAamp DNA Blood Mini Kit (QIAGEN, Hilden, Germany). DNA was diluted in DEPC-treated water (Ambion, Foster City, CA, USA) to a concentration of 10-20 ng/μl. Diluted DNA was bisulfite converted using the EZ DNA Methylation Lightning kit (Zymo, Irvine, CA, USA) according to the manufacturer’s instructions. WGBS libraries were prepared using the TruSeq DNA Methylation kit (Illumina, San Diego, CA, USA) with indexed PCR primers and a 14-cycle PCR program. Libraries were sequenced at 4 per lane on the HiSeq4000 (Illumina, San Diego, CA, USA). Sequencing was performed at the Vincent J. Coates Genomics Sequencing Laboratory at UC Berkeley.

Alignment: Reads without adaptors were aligned using BS-Seeker2 [1], to the hg38 reference sequence, then sorted with samtools. Reads containing adaptors were trimmed, aligned separately, and sorted. Reads with and without adaptors were merged, PCR duplicates were removed, and bam files were converted to sam files using samtools. Sam files were converted to percent methylation bed files using custom Perl scripts. Quality was assessed through analysis of PCR duplicates, mappability, and bisulfite conversion measured as 1 - percent CHH methylation (Additional file 1: Table S1, S10; Additional file 2: Table S2, S11).

Differentially methylated region (DMR) identification: Percent methylation bed files were converted to DSS format using a custom Perl script and DMRs between HC, WD, and DC samples were identified using the bsseq R package [2] and DMRfinder tool [3]. DMRs were further selected for those with a significant 10% methylation difference between groups in liver and 5% methylation difference between groups in blood due to larger sample size. WD-specific DMRs were identified as DMRs between WD and HC samples that overlap DMRs between WD and DC samples, but do not overlap DMRs between WD and HC samples (Additional file 2: Table S3, S4, S12). Genome-wide significant DMRs were identified based on a family-wise error rate (FWER) < 0.05, determined by permuting the samples 1000 times by chromosome, and counting the number of null permutations with equal or better DMRs ranked by number of CpGs and areaStat. Genes were assigned to DMRs in the same manner as the default basal plus extension regulatory domain method used in GREAT, but customized to use gene annotations in the hg38 reference genome [4]. Briefly, regulatory domains were defined for each gene in the NCBI Refseq database with the basal domain as 5 kb upstream to 1 kb downstream of the transcription start site (TSS) and extended in both directions to the nearest gene’s basal domain up to 1 Mb away. A DMR was assigned to a gene if it was overlapping its regulatory domain. DMRs were assigned positions relative to each gene based on overlap with the following priority: TSS, gene body, upstream, and downstream.

Differentially methylated region (DMR)-covariate associations: Smoothed methylation in WD-specific DMRs was obtained using the DMRfinder tool and compared to sample demographic and clinical data using t-test for binary data and linear regression for continuous data (Additional file 2: Table S3, S4, S6, S12, S14). Association of fibrosis stage with liver DMRs as a group was determined through linear regression of the first principal component of smoothed DMR methylation with fibrosis stage in each sample.

Gene ontology enrichment: Enrichment analysis of gene ontology terms were performed using GREAT [4]. A background set of all covered genomic locations assayed for DMRs was used for normalization. DMR and background regions were converted to hg19 using the UCSC Genome Browser liftOver tool and redefined to match a subset of the background regions. GREAT was performed with the default basal plus extension gene regulatory domain definition with curated regulatory domains excluded. Enriched terms had 10 - 2000 annotations and false discovery rate (FDR) *q*-value < 0.05.

ChIP-seq and ChromHMM region enrichment: Enrichment analysis of ChIP-seq and ChromHMM regions was performed using the LOLA R package [5]. Region data was obtained from ENCODE and the Roadmap Epigenomics Project. DMR and background regions were input into LOLA and analysis was performed with DMRs redefined to match a subset of the background regions.

Transcription factor motif enrichment: Enrichment analysis of transcription factor motifs was performed using HOMER [6]. DMR and background regions were input into HOMER and enrichment of known motifs was calculated using exact region sizes and correcting for CpG content.

Cell type-specific hypermethylated promoter analysis: Genes with liver cell type-specific promoters were obtained from a previous MeDIP-ChIP study, which identified promoters specifically methylated in sorted populations of hepatocytes (HEP), hepatic stellate cells (HSC), and liver sinusoidal epithelial cells (LSEC) from human liver [7]. Promoter regions were defined in the hg38 reference genome as 3 kb upstream to 1 kb downstream of the TSS, based on the regions covered on the promoter array used in the study. These regions were overlapped with each set of liver DMRs identified in this paper. Percent methylation in each of the cell type-specific promoters was determined for each of the liver samples from the WGBS data as the number of methylated reads divided by total reads at CpGs in each region. Promoters were subset for only those with at least one read in every sample. Percent methylation was compared between sample groups used to identify liver DMRs. Significance was assessed with percent methylation as the dependent variable and diagnosis as the independent variable using one-way ANOVA followed by Tukey’s post hoc test (Additional file 2: Table S9).

Human-mouse WD DMR gene overlap: Mouse *Atp7b* DMR genes were obtained from a previous study using WGBS to compare methylation in fetal liver at embryonic day 17 between *Atp7b* loss-of-function tx-j mice and wild-type C3H mice [8]. This study used GREAT with the default parameters to annotate DMRs with genes from the mm10 reference genome. Mouse DMR genes were converted to human orthologs using the biomaRt R package [9]. Human orthologs to the mouse DMR genes were overlapped with human DMR genes from the current study with the GeneOverlap R package [10]. Significance was assessed by Fisher’s exact test with the number of background region genes in all human DMR comparisons as the total genome size (Additional file 2: Table S13).

Drug target enrichment: Drug gene annotations were obtained from the Drug Gene Interaction Database [11], and known interacting genes for each drug were overlapped with the liver and blood WD DMR genes using the GeneOverlap R package [10]. Significance was assessed using Fisher’s exact test, using the number of background region genes in all DMR comparisons as the total genome size (Additional file 2: Table S8).

Machine Learning with AdaBoost: We sought to build and independently validate machine learning classifiers with DMR methylation that differentiate neurologic and hepatic WD using training and testing sets in Python. Several packages, including pandas [12], numpy [13], sklearn [14], and matplotlib [15] were used in the processing. 2142 DMRs were used as features for each sample to do machine learning. We used the AdaBoost (Adapting Boosting) classifier with default settings (algorithm='SAMME.R', base_estimator=None, learning_rate=1.0, n_estimators=50, random_state=None) to train on the training set and predict WD phenotype in the testing set [16, 17, 18]. Precision was defined as 1 – the difference between AdaBoost predicted value and real value for the testing set. Predicted value from AdaBoost was extracted out between 0 and 1, if the value is less than 0.5, the AdaBoost model will predict as 0 (neurologic WD) and if the value is more than 0.5, the AdaBoost model will predict as 1 (hepatic WD). After feature selection on the AdaBoost model, 44 out of 2142 DMRs had feature importance greater than zero and were used in the model [16]. The same AdaBoost model was run with only those 44 features, and the precision and predicted value were the same as with the full feature set (Additional file 2: Table S15).

Code Availability: All code for WGBS data analysis is available on GitHub [19].

*Mouse Model Study Methods*

Mice and diets: C3HeB/FeJ (C3H) and C3He-Atp7b<tx-J>/J (tx-j) mouse colonies were maintained according to criteria outlined in the 2011 "Guide for the Care and Use of Laboratory Animals Eighth Edition," published by the National Academy of Sciences, with a light cycle of 14 h light/10 h dark, 20-23°C, and 40-65% relative humidity. Food and deionized water were given ad libitum; C3H were maintained on LabDiet 5001 chow (Purina Lab, St. Louis, MO) and tx-j on Teklad 2020 chow (Envigo, Madison, WI). Mice were conventionally group-housed with 3-4 mice per cage on a mixture of TEK-Fresh (Teklad, Indianapolis, IN) and PAPERCHIP (Shepard Specialty Papers, Watertown, TN) bedding. All protocols were reviewed and approved by the UC Davis Institutional Animal Care and Use Committee, and followed the guidelines of the American Association for Accreditation of Laboratory Animal Care. Since homozygous tx-j mouse milk is copper-deficient and cannot support neonatal growth and development beyond 10-14 days, tx-j mice were produced from a heterozygous cross to avoid cross-fostering pups for survival. Pups from the heterozygous cross were genotyped via Taqman allelic discrimination assay by the Mouse Biology Program at UC Davis. All pups were weaned between 21-28 days of age. Non-fasted male and female mice were anesthetized via isoflurane at 24 weeks of age. Approximately 1 mL of blood was collected from the retro-orbital sinus followed by euthanasia via cervical dislocation. Livers were harvested and sections from each liver were either placed in formalin for subsequent blocking in paraffin or flash-frozen in liquid nitrogen and stored at -80°C for further analysis.

RNA isolation from mouse liver: Total RNA was isolated from mouse liver using the AllPrep DNA/RNA Mini Kit (QIAGEN, Hilden, Germany). Concentration and purity of samples was determined by a Nanodrop spectrophotometer; RNA integrity was evaluated by agarose gel electrophoresis. Total RNA was stored at -80°C until further use.

cDNA synthesis and real-time PCR: Five µg of total RNA was used for reverse transcription following the vendor-provided protocol for the SuperScript III First-Strand cDNA Synthesis kit (Invitrogen, Carlsbad, CA). Primers for mouse cDNA sequences were designed using the free online application Primer 3 [20] and blasted against the mouse genome using NCBI Nucleotide BLAST to check primer specificity [21]. The amplification efficiency (E) of all assays was calculated from the slope of a standard curve generated via 10-fold serial dilution of pooled control cDNA using the formula E = 10(-1/slope) -1. Amplicon specificity was evaluated via in-software melt curve analysis as well as agarose gel electrophoresis of the PCR product. Primer sequences are shown in Additional file 1: Table S16.

**Additional Figures**

**
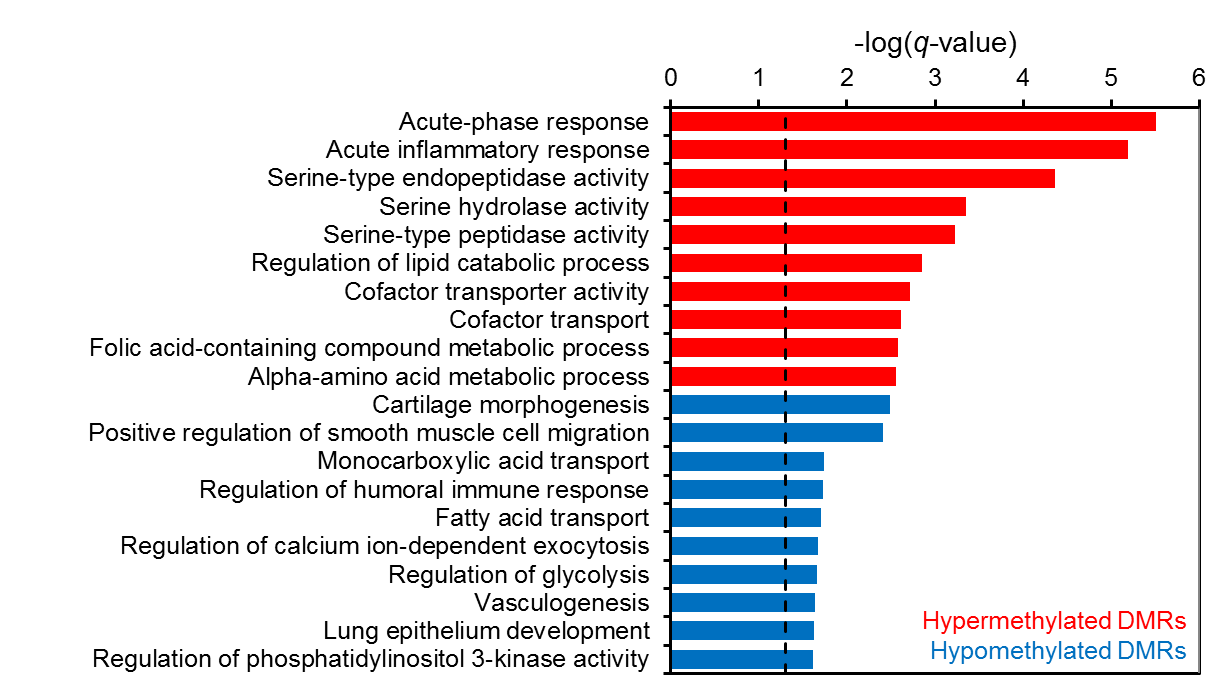
**

**Additional Fig. S1. Results from GREAT functional enrichment analysis of WD-specific liver DMRs compared to background.**

WD-specific liver DMRs are enriched for genes involved in immune response, lipid metabolism, and folic acid metabolism. DMRs differentiating WD from both healthy control and disease control, but not disease control from healthy control, were identified. Hypermethylated DMRs have higher methylation in WD, while hypomethylated DMRs have lower methylation in WD. Top 10 terms from gene ontology databases for hypermethylated or hypomethylated DMRs with FDR *q* < 0.05 are shown, with a dotted line indicating the significance threshold. For this and all subsequent Additional figures: WD, Wilson disease; DMR; differentially methylated region; FDR, false discovery rate.

**
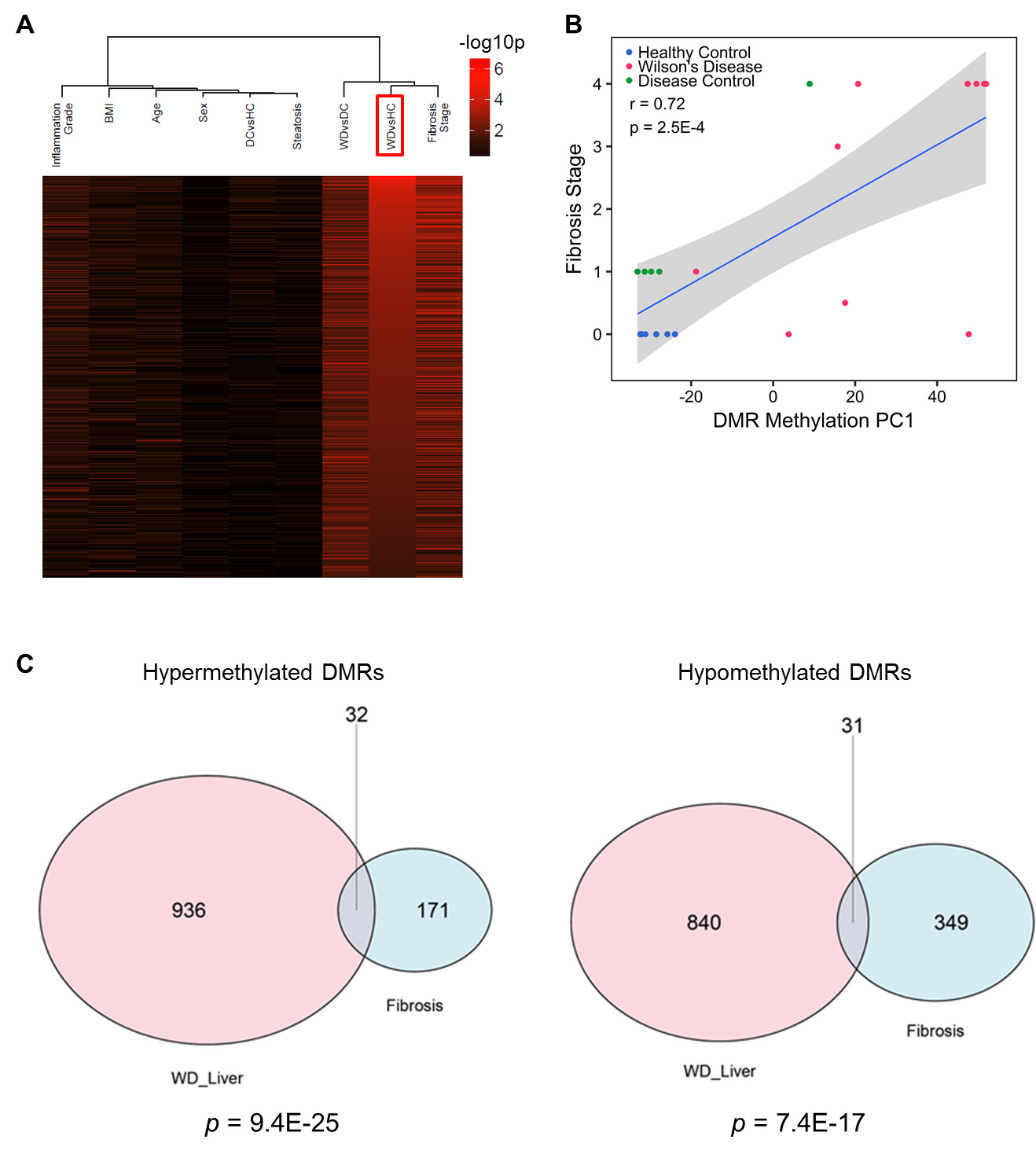
**

**Additional Fig. S2.** **Methylation in WD-specific liver DMRs is associated with diagnosis and fibrosis stage, but not other biological variables.**

(A) Heatmap of biological variables using association -log_10_(p-value) with methylation in WD-specific liver DMRs. (B) Plot of first principal component of the methylation in WD-specific liver DMRs compared to fibrosis stage. Shaded area indicates 95% confidence interval. (C) Overlap of WD-specific liver DMRs compared to fibrosis-associated DMRs [22] by direction of methylation change. Overlap significance was calculated by hypergeometric test.


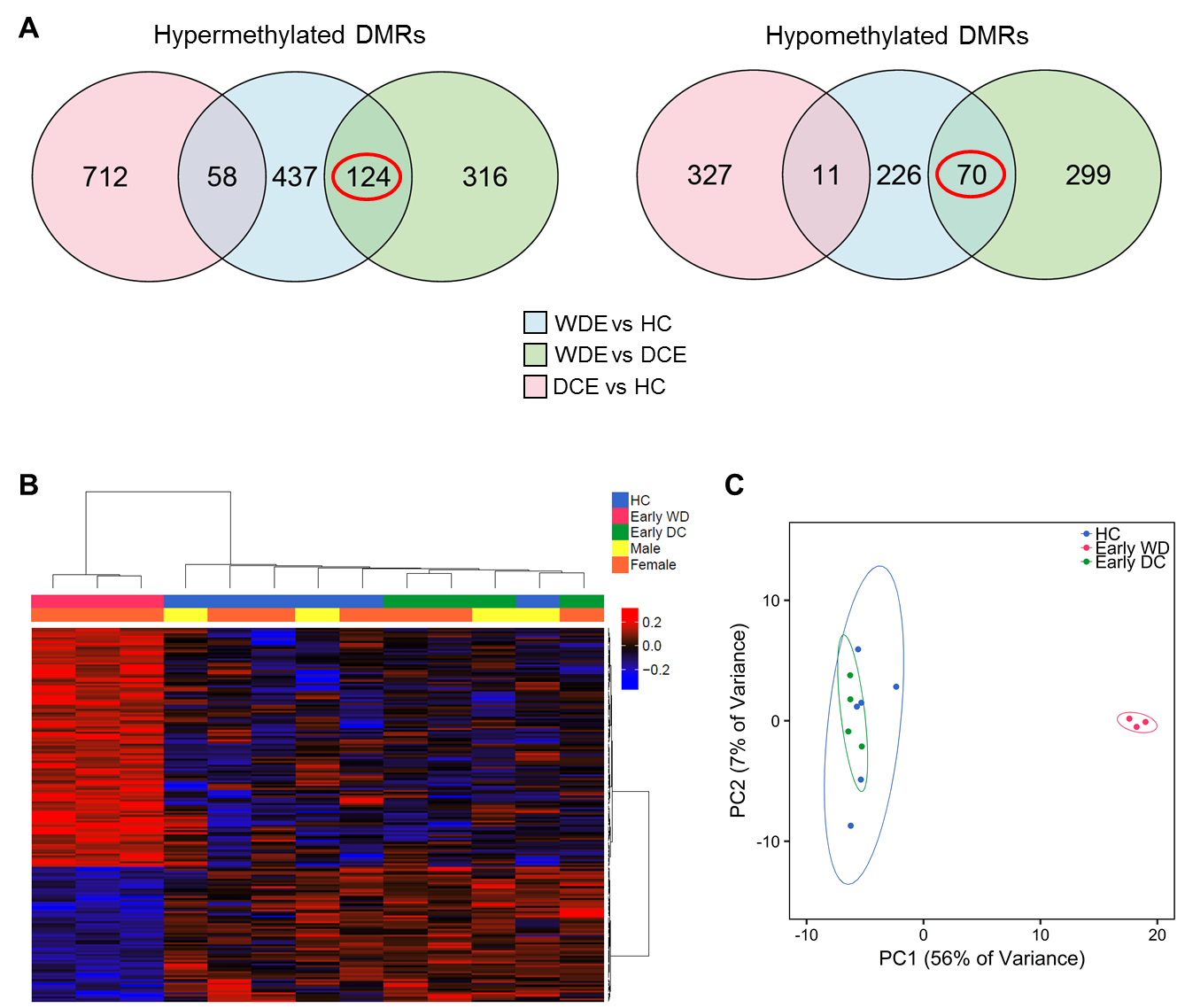


**Additional Fig. S3.** **DMRs in liver distinguish early-stage WD from early-stage controls.**

(A) WGBS was performed, and DMRs that differentiate early-stage WD (WDE) liver samples from both HC and early-stage DC (DCE) liver samples, but not DCE from HC liver samples, were identified (WDE *n*=3, HC *n*=6, DCE *n*=4). Hypermethylated DMRs have higher methylation in WDE, while hypomethylated DMRs have lower methylation in WDE. (B) Heatmap of HC, WDE, and DCE samples using methylation in WDE-specific liver DMRs. Percent methylation for each sample relative to the mean methylation at each DMR is plotted. (C) Principal component analysis using methylation in WDE-specific liver DMRs. Ellipses show 95% confidence intervals.

**
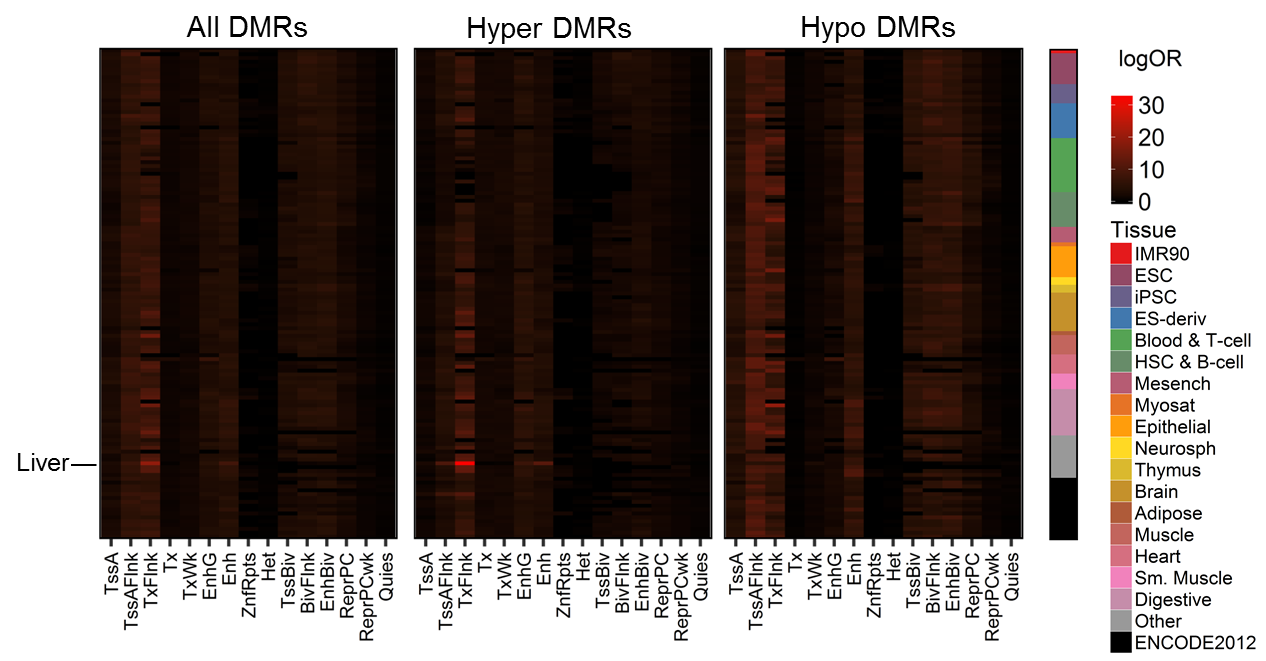
**

Odds Ratio

**Additional Fig. S4.** **WD-specific liver DMRs are enriched in liver enhancers and flanking active liver promoters with direction-specific effect.**

WD liver DMRs were overlapped with chromatin states from the Epigenome Roadmap using LOLA and the odds ratio was plotted for all tissues.

**
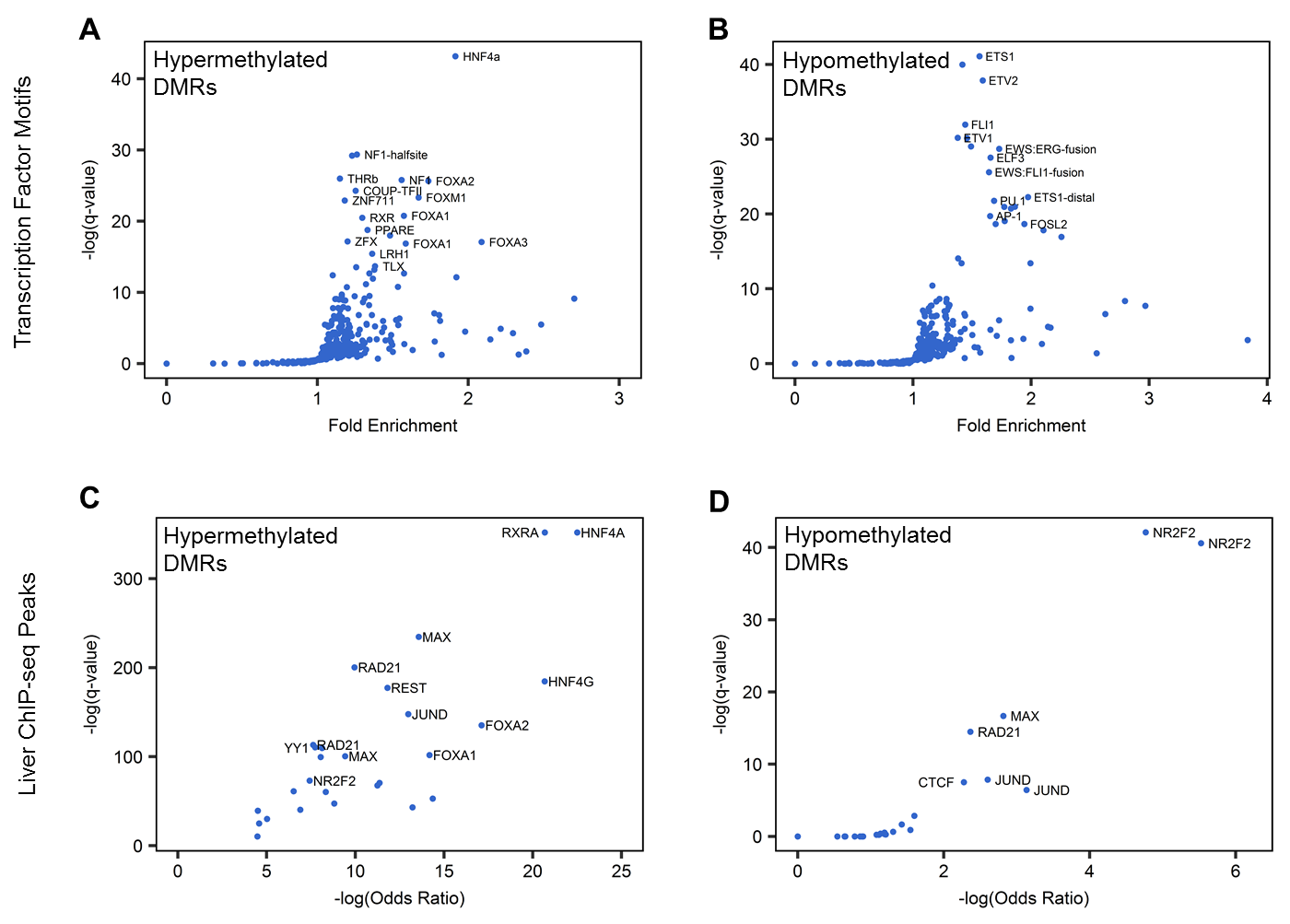
**

**Additional Fig. S5.** **WD-associated liver hypermethylated and hypomethylated DMRs are enriched in different liver-associated transcription factor binding sites.**

(A, B) WD liver DMR sequences were tested for enriched known transcription factor motifs using HOMER. (C, D) WD liver DMRs were tested for enrichment with transcription factor ChIP-seq peaks in liver using LOLA. Volcano plots are shown for (A, C) hypermethylated DMRs and (B, D) hypomethylated DMRs.

**
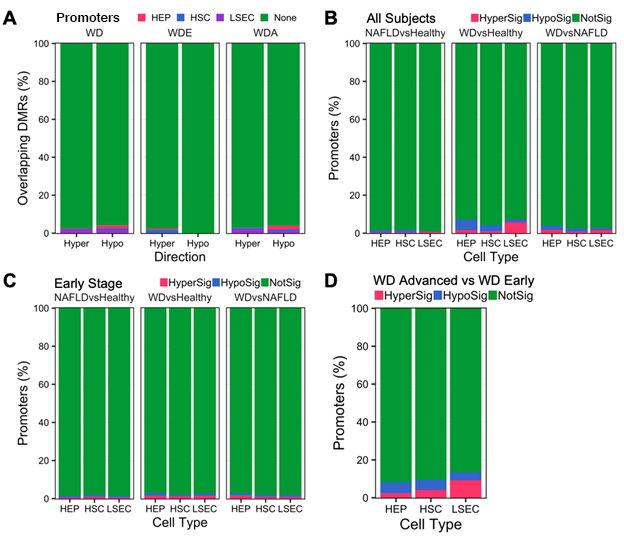
**

**Additional Fig. S6.** **WD-associated liver methylation is not influenced by cell-type changes.**

(A) Liver DMRs from WD-specific (WD), WD Early-specific (WDE), and WD Advanced vs Early (WDA) DMR sets were overlapped with human liver cell type-specific hypermethylated promoters identified previously [7]. Percent of overlapping DMRs for each set is plotted by direction for hepatocytes (HEP), hepatic stellate cells (HSC), and liver sinusoidal epithelial cells (LSEC). (B-D) Percent methylation at cell type-specific hypermethylated promoters was compared between sample groups used to identify liver DMRs for (B) WD, (C) WDE, and (D) WDA. Significance was assessed using one-way ANOVA followed by Tukey’s post hoc test (Sig: *p* < 0.05). Percent of specific hypermethylated promoters with nominally significant differences is plotted by direction for each cell type and comparison.

**
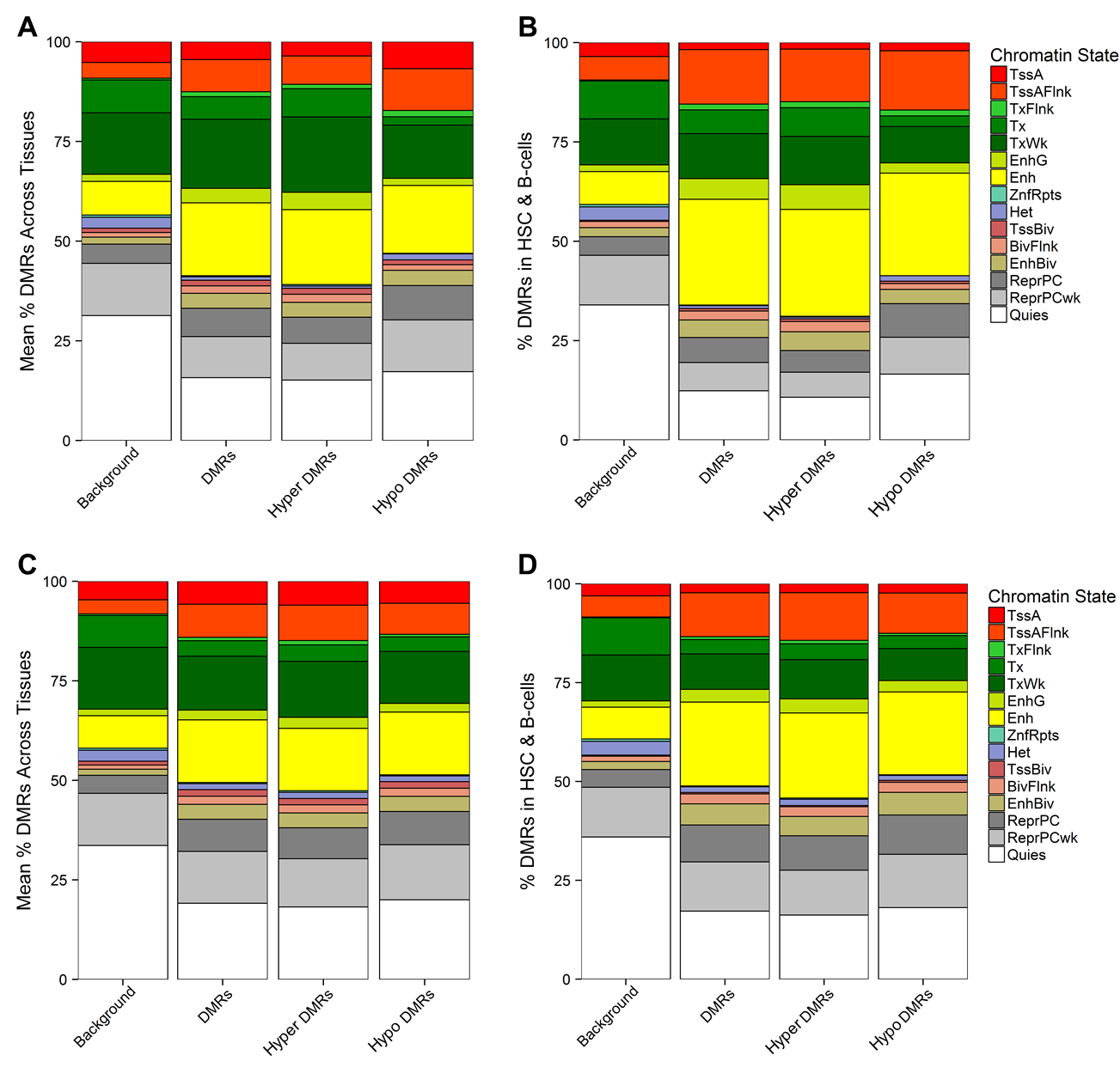
 Additional Fig. S7.** **WD-specific DMRs in blood are enriched in HSC and B-cell enhancers, while WD Hepatic vs Neurologic DMRs are moderately enriched in bivalent enhancers.**

(A, B) WD-specific blood DMRs were overlapped with chromatin states from the Epigenome Roadmap using LOLA. (C, D) WD Hepatic vs Neurologic blood DMRs were overlapped with chromatin stats from the Epigenome Roadmap using LOLA. The percent of DMRs and background regions overlapping each state was plotted as (A, C) the mean overlap for all tissues or (B, D) overlap for HSC & B-cells.

**
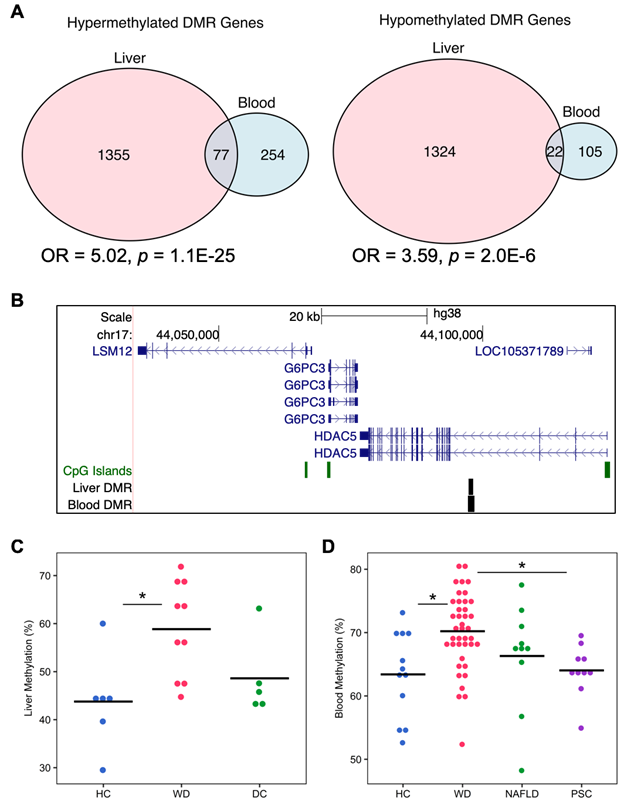
Additional Fig. S8.** **WD-specific DMRs in liver and blood overlap by gene.**

(A) Genes linked by GREAT to WD-specific DMRs in liver and blood were overlapped by direction. Hypermethylated DMRs have higher methylation in WD, while hypomethylated DMRs have lower methylation in WD. Significance testing was done by Fisher’s Exact Test. (B) Genome Browser view of overlapping WD-specific DMRs at *HDAC5* in liver and blood. (C, D) Methylation at WD-specific liver DMR at *HDAC5* in (C) liver and (D) blood samples. Bars indicate mean methylation for each group (* *p* < 0.05 by ANOVA followed by Tukey’s post hoc test). Significant overall effect of diagnosis on methylation in liver (*p* = 0.018) and blood (*p* = 0.003).


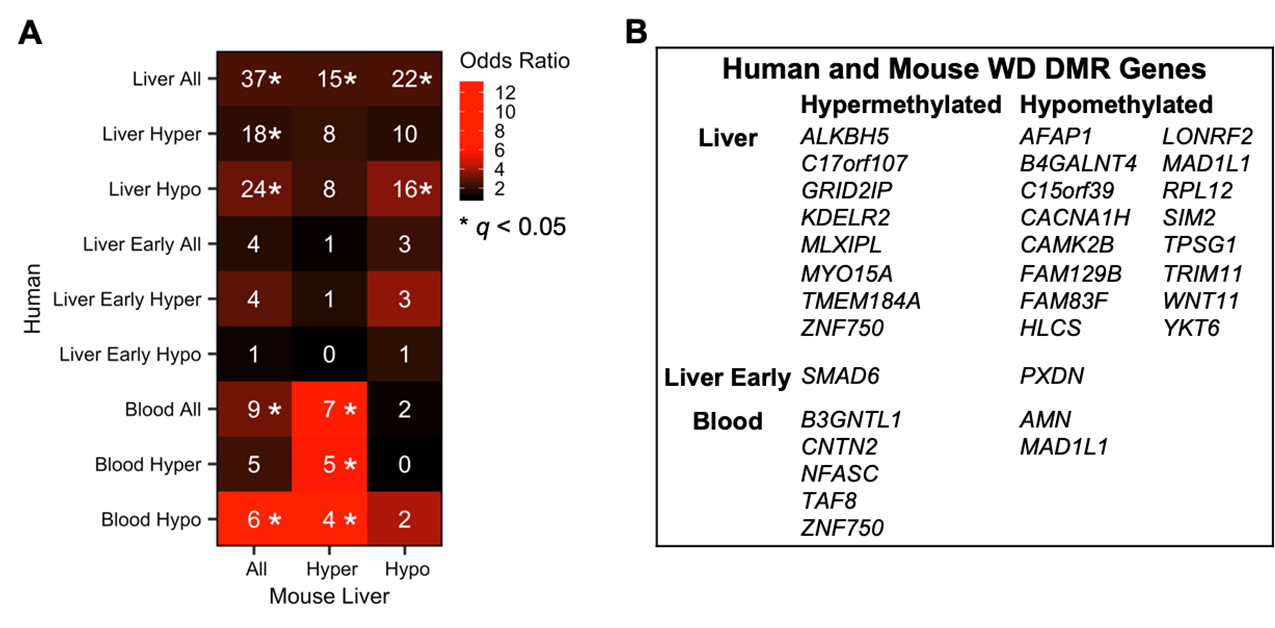


**Additional Fig. S9.** **Human and mouse WD DMR genes overlap within and across tissues.**

(A, B) Genes linked by GREAT to WD-specific DMRs in liver and blood and WDE-specific DMRs in liver were overlapped with orthologous genes linked to *Atp7b* loss of function DMRs in tx-j versus C3H mouse fetal liver [8]. (A) The heatmap displays the number of overlapping genes and is colored by the odds ratio. Significance was determined with Fisher’s exact test and *p*-values were adjusted using the false discovery rate method (* *q* < 0.05). (B) Human DMR genes overlapping with orthologous mouse DMR genes with differential methylation in the same direction are shown for each comparison.

**Additional Tables**

See Additional file 2 for Additional Tables S2-S4, S6, S8, S9, and S11-S15.

**Additional Table S1. Summary statistics for clinical features and WGBS quality control of subjects providing liver samples.**

|  | **Diagnosis** | | |  | |  | |
| --- | --- | --- | --- | --- | --- | --- | --- |
|  | **Healthy (n = 6)** | **NAFLD (n = 5)** | **WD (n = 10)** | | ***p*-value^a^** | | ***q*-value^b^** |
| Sex | 3M/3F | 2M/3F | 5M/5F | | 1.00 | | 1.00 |
| Age (years) | 48.7 ± 15.1 | 47.6 ± 20.4 | 37.2 ± 9.4 | | 0.23 | | 0.42 |
| BMI^c^ | 39.6 ± 11.6 | 40.0 ± 8.1 | 31.6 ± 2.6 | | 0.25 | | 0.42 |
| Fibrosis | 0 ± 0 | 1.6 ± 1.3 | 2.5 ± 1.8 | | **0.01** | | 0.09 |
| Steatosis^d^ | 0 ± 0 | 1.4 ± 0.9 | 0.8 ± 0.8 | | **0.02** | | 0.09 |
| Inflammation^e^ | 0.2 ± 0.4 | 1.0 ± 0.7 | 1.3 ± 1.4 | | 0.12 | | 0.35 |
| Coverage | 1.5 ± 0.4 | 1.5 ± 0.3 | 1.3 ± 0.2 | | 0.15 | | 0.35 |
| Mappability (%) | 72.5 ± 1.3 | 73.5 ± 0.7 | 72.8 ± 1.9 | | 0.57 | | 0.62 |
| mCG (%) | 74.1 ± 1.7 | 74.3 ± 0.7 | 74.4 ± 0.7 | | 0.87 | | 0.87 |
| mCHG (%) | 1.7 ± 0.1 | 1.8 ± 0.1 | 1.7 ± 0.1 | | 0.08 | | 0.33 |
| mCHH (%) | 1.3 ± 0.1 | 1.3 ± 0.1 | 1.3 ± 0.1 | | 0.39 | | 0.50 |
| Conversion Efficiency (%) | 98.7 ± 0.1 | 98.7 ± 0.1 | 98.7 ± 0.1 | | 0.39 | | 0.50 |
| PCR Duplicates (%) | 23.6 ± 4.8 | 24.4 ± 3.6 | 21.8 ± 3.1 | | 0.42 | | 0.50 |

NAFLD, Non-alcoholic fatty liver disease; WD Wilson disease. Values are expressed as mean ± SD. *p* < 0.05 considered significant (bolded). ^a^*p*-values from Fisher’s exact test for categorical variables and one-way ANOVA for continuous variables; ^b^*q*-values determined by false discovery rate method; ^c^Frequency missing = 5 in WD; ^d^Frequency missing = 5 in WD; ^e^Frequency missing = 4 in WD.

**Additional Table S2. Clinical features and WGBS quality control of subjects providing liver samples.**

**Additional Table S3. WD-specific liver DMR analysis.**

**Additional Table S4. Early-stage WD-specific liver DMR analysis.**

**Additional Table S5. Transcript levels for selected mouse liver genes.**

|  | **C3H** | | | **tx-j** | | | ***q*-value** | | |
| --- | --- | --- | --- | --- | --- | --- | --- | --- | --- |
| Gene | M + F (n=22) | M (n=10) | F (n=12) | M + F (n=22) | M (n=11) | F (n=11) | M + F | M | F |
| *Foxa1* | 1.10 ± 0.57 | 1.33 ± 0.72 | 0.91 ± 0.34 | 0.70 ± 0.18 | 0.69 ± 0.24 | 0.70 ± 0.11 | **5.39E-03** | **2.28E-02** | 6.93E-02 |
| *Gata6* | 1.02 ± 0.21 | 0.99 ± 0.24 | 1.05 ± 0.19 | 1.68 ± 0.24 | 1.55 ± 0.16 | 1.82 ± 0.25 | **1.24E-10** | **3.09E-05** | **6.12E-07** |
| *Hdac5* | 1.01 ± 0.17 | 0.96 ± 0.19 | 1.06 ± 0.15 | 1.39 ± 0.29 | 1.21 ± 0.25 | 1.57 ± 0.21 | **2.15E-05** | **1.96E-02** | **9.41E-06** |
| *Mafb* | 1.06 ± 0.33 | 1.01 ± 0.30 | 1.09 ± 0.37 | 0.72 ± 0.17 | 0.65 ± 0.12 | 0.79 ± 0.19 | **3.16E-04** | **5.39E-03** | **2.26E-02** |
| *Nacc2* | 1.00 ± 0.10 | 0.98 ± 0.07 | 1.03 ± 0.12 | 0.82 ± 0.11 | 0.77 ± 0.10 | 0.87 ± 0.09 | **4.23E-06** | **9.94E-05** | **4.02E-03** |
| *Pcx* | 1.01 ± 0.13 | 0.94 ± 0.08 | 1.06 ± 0.13 | 0.75 ± 0.13 | 0.65 ± 0.08 | 0.85 ± 0.09 | **3.23E-07** | **6.90E-07** | **3.16E-04** |
| *Pmpca* | 1.01 ± 0.13 | 1.00 ± 0.09 | 1.01 ± 0.16 | 1.28 ± 0.18 | 1.15 ± 0.09 | 1.41 ± 0.13 | **2.71E-06** | **2.80E-03** | **4.47E-06** |
| *Pnpla7* | 1.01 ± 0.16 | 1.11 ± 0.13 | 0.93 ± 0.13 | 1.31 ± 0.29 | 1.07 ± 0.19 | 1.54 ± 0.12 | **3.16E-04** | 5.63E-01 | **1.23E-09** |
| *Tspan9* | 1.01 ± 0.14 | 0.93 ± 0.09 | 1.08 ± 0.14 | 1.60 ± 0.30 | 1.51 ± 0.35 | 1.69 ± 0.21 | **1.77E-08** | **3.16E-04** | **1.33E-06** |
| *Vtn* | 1.01 ± 0.17 | 1.03 ± 0.15 | 1.00 ± 0.19 | 0.79 ± 0.11 | 0.77 ± 0.10 | 0.81 ± 0.12 | **2.15E-05** | **6.53E-04** | **9.79E-03** |

C3H = wild-type control, tx-j = mouse model of Wilson disease.

Values are expressed as mean ± SD. *p*-value = Student’s t-test comparison of C3H vs. tx-j within the same sex group; FDR *q* < 0.05 considered significant (bolded).

**Additional Table S6. Advanced- vs early-stage WD liver DMR analysis.**

**Additional Table S7. Genome-wide significant Wilson disease liver progression DMRs and associated genes.**

| Chr | Start | End | CpGs | Methylation  Difference | FWER | Gene | Distance to  TSS (kb) | Position |
| --- | --- | --- | --- | --- | --- | --- | --- | --- |
| chr4 | 1170728 | 1171806 | 64 | -23% | < 0.001 | *CTBP1-AS* | -38.3 | upstream |
|  |  |  |  |  |  | *TMED11P* | -47.6 | upstream |
| chr9 | 34371150 | 34371812 | 58 | -12% | < 0.001 | *KIAA1161* | 5.1 | exon |
|  |  |  |  |  |  | *KIF24* | -42.0 | upstream |
| chr4 | 39446853 | 39447434 | 45 | -34% | 0.008 | *LIAS* | -11.6 | upstream |
|  |  |  |  |  |  | *MIR5591* | 34.9 | downstream |
| chr18 | 45837574 | 45837967 | 52 | -35% | 0.008 | *SIGLEC15* | 12.0 | exon |
|  |  |  |  |  |  | *EPG5* | 129.4 | downstream |
| chr3 | 49685991 | 49687155 | 42 | -29% | 0.017 | *RNF123* | -2.3 | upstream |
| chr13 | 113109706 | 113110882 | 52 | -31% | 0.017 | *F7* | 3.9 | exon |
|  |  |  |  |  |  | *F10* | -11.9 | upstream |
| chr9 | 133702342 | 133703045 | 38 | -29% | 0.025 | *SARDH* | 36.9 | exon |
|  |  |  |  |  |  | *DBH-AS1* | -44.9 | upstream |
| chr14 | 103102252 | 103102814 | 66 | -16% | 0.025 | *EXOC3L4* | 2.1 | exon |
|  |  |  |  |  |  | *TNFAIP2* | -23.5 | upstream |
| chr3 | 157120258 | 157120785 | 46 | 28% | 0.025 | *LINC00880* | 2.2 | intron |
|  |  |  |  |  |  | *LINC00881* | 30.4 | downstream |
| chr17 | 42122350 | 42123365 | 44 | 32% | 0.034 | *HSPB9* | 0.0 | TSS |
|  |  |  |  |  |  | *KAT2A* | -1.0 | upstream |
| chr5 | 177402511 | 177403597 | 64 | -31% | 0.034 | *PFN3* | -1.9 | upstream |
| chr9 | 114504332 | 114504715 | 36 | -29% | 0.034 | *WHRN* | 0.7 | exon |
|  |  |  |  |  |  | *AKNA* | -109.9 | upstream |
| chr16 | 57528712 | 57529343 | 42 | 28% | 0.042 | *CCDC102A* | 7.2 | exon |
|  |  |  |  |  |  | *DOK4* | -42.2 | upstream |
| chr21 | 44438468 | 44439050 | 18 | 31% | 0.042 | *TRPM2-AS* | -12.9 | upstream |
|  |  |  |  |  |  | *LRRC3* | -16.4 | upstream |
| chr13 | 113832229 | 113832692 | 17 | 41% | 0.050 | *GAS6-AS1* | 16.6 | intron |
|  |  |  |  |  |  | *GAS6-AS2* | -31.5 | upstream |

FWER, family-wise error rate.

**Additional Table S8. DMR drug target enrichment.**

**Additional Table S9. Liver cell type-specific promoter methylation analysis.**

**Additional Table S10. Summary statistics for clinical features and WGBS quality control of subjects providing blood samples.**

|  | **Diagnosis** | | | | |  | |
| --- | --- | --- | --- | --- | --- | --- | --- |
|  | **Healthy (n = 12)** | **NAFLD (n = 10)** | **PSC (n = 10)** | **WDH (n = 25)** | **WDN (n = 25)** | ***p*-value^a^** | ***q*-value^b^** |
| Sex | 6M/6F | 5M/5F | 5M/5F | 13M/12F | 14M/11F | 1.00 | 1.00 |
| Age (years) | 36.4 ± 12.1 | 51.0 ± 12.5 | 47.2 ± 14.4 | 33.6 ± 11.5 | 35.6 ± 10.9 | **0.001** | **0.002** |
| BMI^c^ | 24.4 ± 3.7 | 33.7 ± 7.2 | 28.1 ± 7.7 | 25.5 ± 3.9 | 24.7 ± 3.8 | **0.0002** | **0.001** |
| Coverage | 1.5 ± 0.2 | 1.6 ± 0.3 | 1.4 ± 0.3 | 1.4 ± 0.4 | 1.3 ± 0.4 | 0.15 | 0.17 |
| Mappability (%) | 71.7 ± 1.2 | 70.9 ± 6.9 | 68.8 ± 8.7 | 71.7 ± 6.4 | 68.4 ± 10.7 | 0.55 | 0.55 |
| mCG (%) | 76.7 ± 0.6 | 77.1 ± 0.8 | 77.1 ± 1.1 | 76.5 ± 0.8 | 76.4 ± 0.8 | 0.08 | 0.11 |
| mCHG (%) | 1.8 ± 0.3 | 2.1 ± 0.2 | 2.2 ± 0.3 | 1.7 ± 0.3 | 1.8 ± 0.4 | **0.001** | **0.002** |
| mCHH (%) | 1.4 ± 0.1 | 1.6 ± 0.1 | 1.6 ± 0.2 | 1.3 ± 0.3 | 1.3 ± 0.3 | **0.01** | **0.02** |
| Conversion Efficiency (%) | 98.6 ± 0.1 | 98.5 ± 0.1 | 98.4 ± 0.2 | 98.7 ± 0.3 | 98.7 ± 0.3 | **0.01** | **0.02** |
| PCR Duplicates (%) | 25.0 ± 2.2 | 27.4 ± 4.3 | 24.4 ± 2.6 | 22.1 ± 8.2 | 21.8 ± 8.0 | 0.15 | 0.17 |

NAFLD, Non-alcoholic fatty liver disease; PSC, Primary sclerosing cholangitis; WDH, Wilson disease – hepatic; WDN, Wilson disease – neurologic. Values are expressed as mean ± SD. *p* < 0.05 considered significant (bolded). ^a^*p*-values from Fisher’s exact test for categorical variables and one-way ANOVA for continuous variables; ^b^*q*-values determined by false discovery rate method; ^c^Frequency missing = 5 in WDH, 5 in WDN.

**Additional Table S11. Clinical features and WGBS quality control of subjects providing blood samples.**

**Additional Table S12. WD-specific blood DMR analysis.**

**Additional Table S13. Human and mouse WD DMR gene overlap analysis.**

**Additional Table S14. Hepatic vs neurologic WD blood DMR analysis.**

**Additional Table S15. Adaboost-selected hepatic vs neurologic WD blood DMRs.**

**Additional Table S16. Real-time PCR primers for selected mouse liver genes.**

| **Gene name** | **Gene** | **Primer** | **Sequence 5' to 3'** | **Exon-exon overlap** |
| --- | --- | --- | --- | --- |
| *Foxa1* | forkhead box A1 | F | CGAGTTTACAGGTCTGTGGCAAT | No |
|  |  | R | GAGCCCCTACTGTCTACTTGGTTTC | No |
| *Gata6* | GATA binding protein 6 | F | CTCCGGTAACAGCAGTGGC | Yes |
|  |  | R | CTGAGGTGGTCGCTTGTGTAGA | No |
| *Hdac5* | histone deacetylase 5 | F | TCGTGGACTGGGATATTCACC | Yes |
|  |  | R | CCACCAACCTCTTCAGGAGC | Yes |
| *Mafb* | v-maf musculoaponeurotic fibrosarcoma oncogene family, protein B (avian) | F | CATTGAGCCAAACAGCCATTC | No |
|  |  | R | GACACACTTGAGAGTTGCAGCG | No |
| *Nacc2* | nucleus accumbens associated 2, BEN and BTB (POZ) domain containing | F | GAATGGCGACAAGCACATGAT | No |
|  |  | R | TGAGCCCTTCTTCTAGCAGCATA | No |
| *Pcx* | pyruvate carboxylase | F | GGCCATGAAGGAGATGCACT | Yes |
|  |  | R | GCTGCCACCTTGATGTCTATGA | No |
| *Pmpca* | peptidase (mitochondrial processing) alpha | F | CAGACCCAAGACAAGTTCGAGA | Yes |
|  |  | R | GCTCCACCAAATCCACCGT | Yes |
| *Pnpla7* | patatin-like phospholipase domain containing 7 | F | ACCGAGTATGAGGAGGAACTGC | No |
|  |  | R | GAAGGCTCATATTCCGAGTCAGAG | Yes |
| *Tspan9* | tetraspanin 9 | F | TCCCACAAGTTGCCAGTTCC | No |
|  |  | R | CGCCATGCTTGCTTTCTGTT | No |
| *Vtn* | vitronectin | F | GAGAGTGGGCTAGGAACCTACAAC | No |
|  |  | R | TGACTCGGTAGTATTTGTCTCCAGAG | Yes |

**Additional References**

1. Guo W, Fiziev P, Yan W, Cokus S, Sun X, Zhang MQ, et al. BS-Seeker2; a versatile aligning pipeliine for bisulfite sequencing data. BMC Genomics. 2013;14:774.
2. Hansen KD, Langmead B, Irizarry RA. BSmooth; from whole genome bisulfite sequencing reads to differentially methylated regions. Genome Biol. 2012;13:R83.
3. Mordaunt CE. DMRfinder. R workflow. <https://github.com/cemordaunt/DMRfinder>. Accessed 20 Dec 2018.
4. McLean C, Bristor D, Hiller M, Clarke SL, Schaar BT, Lowe CB, et al. GREAT improves functional interpretation of cis-regulatory regions. Nat Biotechnol. 2010;28:495-501.
5. Sheffield NC, Bock C. LOLA: enrichment analysis for genomic region sets and regulatory elements in R and Bioconductor. Bioinformatics. 2016;32:587-9.
6. Heinz S, Benner C, Spann N, Bertolino E, Lin YC, Laslo P, et al. Simple combinations of lineage-determining transcription factors prime cis-regulatory elements required for macrophage and B-cell identities. Mol Cell. 2010;38:576-89.
7. Taghdouini AE, Sorensen AL, Reiner AH, Coll M, Verhulst S, Mannaerts I, et al. Genome-wide analysis of DNA methylation and gene expression patterns in purified, uncultured human liver cells and activated hepatic stellate cells. Oncotarget. 2015;6(29):26729-45.
8. Mordaunt CE, Shibata NM, Kieffer DA, Czlonkowska A, Litwin T, Weiss KH, et al. Epigenetic changes of the thioredoxin system in the tx-j mouse model and in patients with Wilson disease. Hum Mol Genet. 2018; 27(22):3854-69.
9. Durinck S, Spellman P, Birney E, Huber W. Mapping identifiers for the integration of genomic datasets with the R/Bioconductor package biomaRt. Nat Protoc. 2009;4:1184–1191.
10. Shen L and Sinai M. GeneOverlap: Test and visualize gene overlaps. R package version 1.18.0. 2018; <http://shenlab-sinai.github.io/shenlab-sinai/>. Accessed 23 Feb 2018.
11. Cotto KC, Wagner AH, Feng YY, Kiwala S, Coffman AC, Spies G, et al. DGIdb 3.0: a redesign and expansion of the drug-gene interaction database. Nucleic Acids Res. 2017;46:D1068-73.
12. Mckinney W. pandas: a foundational Python library for data analysis and statistics. Python for High Performance and Scientific Computing. 2011;1-9.
13. Oliphant TE. Guide to NumPy. Trelgol Publishing; 2006.
14. Buitinck L, Louppe G, Blondel M, Pedregosa F, Mueller A, Grisel O, et al. API design for machine learning software: experiences from the scikit-learn project. European Conference on Machine Learning and Principles and Practices of Knowledge Discovery in Databases. 2013: hal-00856511.
15. Hunter JD. Matplotlib: a 2D graphics environment. Comput Sci Eng. 2007;9:90-5.
16. Wang R. AdaBoost for feature selection, classification and its relation with SVM, a review. Phys Procedia. 2012;25:800–7.
17. Solomatine DP and Shrestha DL. AdaBoost.RT: a boosting algorithm for regression problems. IEEE International Joint Conference on Neural Networks. 2004;2:1163–8.
18. Gosztolya G, Busa-Fekete R, and Tóth L. Detecting Autism, emotions and social signals using AdaBoost. Interspeech. 2013;220-4.
19. Mordaunt CE. Code Availability. <https://github.com/cemordaunt/WilsonDiseaseEpigenome>. Accessed 15 Sep 2018.
20. Primer 3 (v.0.4.0). <http://bioinfo.ut.ee/primer3-0.4.0/>. Accessed 22 Feb 2018.
21. NCBI Nucleotide BLAST. <http://blast.ncbi.nlm.nih.gov/Blast.cgi>. Accessed 22 Feb 2018.
22. Hotta K, Kitamoto T, Kitamoto A, Ogawa Y, Honda Y, Kessoku T, et al. Identification of the genomic region under epigenetic regulation during non-alcoholic fatty liver disease progression. Hepatol Res. 2018;48:E320-34.
